# Analysis of the effects of importin α1 on the nuclear translocation of IL-1α in HeLa cells

**DOI:** 10.1038/s41598-024-51521-w

**Published:** 2024-01-15

**Authors:** Akiko Yamada, Kiyotaka Wake, Saya Imaoka, Mitsuru Motoyoshi, Takenori Yamamoto, Masatake Asano

**Affiliations:** 1 Department of Pathology, Nihon University School of Dentistry, 1-8-13, Kanda-Surugadai, Chiyoda-ku, Tokyo, 101-8310 Japan; 2 Division of Immunology and Pathobiology, Dental Research Center, Nihon University School of Dentistry, 1-8-13, Kanda-Surugadai, Chiyoda-ku, Tokyo, 101-8310 Japan; 3 Department of Orthodontics, Nihon University School of Dentistry, 1-8-13, Kanda-Surugadai, Chiyoda-ku, Tokyo, 101-8310 Japan; 4 Division of Oral Structural and Functional Biology, Nihon University Graduate School of Dentistry, 1-8-13, Kanda-Surugadai, Chiyoda-ku, Tokyo, 101-8310 Japan; 5 Division of Clinical Research, Dental Research Center, Nihon University School of Dentistry, 1-8-13, Kanda-Surugadai, Chiyoda-ku, Tokyo, 101-8310 Japan; 6 Division of Molecular Target and Gene Therapy Products, National Institute of Health Sciences, 3-25-26, Tonomachi, Kawasaki-ku, Kawasaki-shi, Kanagawa, 210-9501 Japan; 7 Institute for Genome Research, Tokushima University, Kuramotocho-3, Tokushima, 770-8503 Japan

**Keywords:** Biochemistry, Cell biology, Molecular biology

## Abstract

Interleukin-1α (IL-1α), a cytokine released by necrotic cells, causes sterile inflammation. On the other hand, IL-1α is present in the nucleus and also regulates the expression of many proteins. A protein substrate containing a classical nuclear localization signal (cNLS) typically forms a substrate/importin α/β complex, which is subsequently transported to the nucleus. To the best of our knowledge, no study has directly investigated whether IL-1α—which includes cNLS—is imported into the nucleus in an importin α/β-dependent manner. In this study, we noted that all detected importin α subtypes interacted with IL-1α. In HeLa cells, importin α1-mediated nuclear translocation of IL-1α occurred at steady state and was independent of importin β1. Importin α1 not only was engaged in IL-1α nuclear transport but also concurrently functioned as a molecule that regulated IL-1α protein level in the cell. Furthermore, we discussed the underlying mechanism of IL-1α nuclear translocation by importin α1 based on our findings.

## Introduction

Interleukin-1α (IL-1α), a member of the IL-1 family that is extensively present in mesenchymal-derived tissues and epithelial cells^1^. The precursor of IL-1α has a molecular weight of approximately 33 kDa and is cleaved by proteases into a 17-kDa mature form and a 16-kDa N-terminal form called a propiece^2,3^. However, most IL-1α is present in its precursor form, the majority of which is in intracellular proteins or membrane forms^4,5^. The activity of the precursor form of IL-1α is inhibited intracellularly by IL-1 receptor type 2, a decoy receptor^6,7^. When cells undergo necrosis, the precursor form of IL-1α is immediately released extracellularly, acting as an “alarmin” and informing the loss of membrane integrity to nearby cells^1^. Extracellularly released IL-1α precursor binds to IL-1 receptor type 1 (IL-1R1) on neighboring cells, stimulating the production of cytokines such as IL-6^8^, and promoting tissue inflammation^4,5^.

Furthermore, IL-1α is known for its role as a dual-function cytokine; it has a nuclear localization signal (NLS) sequence^9^ and can be transported to the nucleus^10^. In the nucleus, IL-1α activates NF-κB and AP-1 in an IL-1R1-independent manner, promoting the production of inflammatory cytokines such as IL-6 and IL-8^11^. In addition, IL-1α was reported to interact with histone acetyl transferase complexes in vitro^12,13^, with mRNA splicing-related proteins, promoting apoptosis of malignant tumor cells^14^, and that IL-1α expression lowers cell growth rates and migratory potential in vascular endothelial cells^15,16^. These findings suggested that IL-1α is involved in regulation of the expression of various proteins in the nucleus. Therefore, regulating the nuclear translocation of IL-1α is effective for the control of intranuclear IL-1α-related diseases.

The mechanisms of IL-1α nuclear localization are unknown. Several findings regarding IL-1α nuclear transport were previously reported. Cohen et al*.* discovered that acetylation of IL-1α at Lys82 promoted the nuclear localization of IL-1α during genotoxic stress in the murine macrophage cell line RAW 264.7^17^. Yin et al. found that HAX-1 interacted with three domains of the N-terminus of IL-1α, including the NLS-containing domain, in HEK293 cells using an immunoprecipitation assay^18^. According to Kawaguchi et al., HAX-1 knockdown in systemic sclerosis fibroblasts reduced nuclear IL-1α levels^19^. However, the mechanism through which Lys82 of IL-1α and HAX-1 are involved in the nuclear localization of IL-1α requires further elucidation. On the contrary, the most prevalent nuclear localization signal, namely the classical NLS (cNLS; KVLKKRRL)^9^, is found in IL-1α^20,21^. In the cytoplasm, cNLS-containing molecule and importin α, a cargo receptor, and importin β1, a carrier molecule, form a complex, passing through the nuclear–pore complex by utilizing the gradient of the small G proteins Ran-GDP and Ran-GTP^22–24^. Luheshi et al*.* reported that the nuclear translocation of IL-1α is Ran-dependent upon analyzing COS-7 cells coexpressing IL-1α with RanQ69L, a dominant-negative isoform of Ran lacking the ability to hydrolyze GTP^25^. Sahni et al*.* studied the influence of disease-associated mutations on the protein interactome using a comprehensive yeast two-hybrid (Y2H) screening assay, which revealed that importin α7 interacts with IL-1α^26^. The findings that IL-1α is transported to the nucleus via Ran-GDP/GTP gradient and that IL-1α interacts with the importin α family in yeast cells suggest that IL-1α is transported to the nucleus in an importin α dependent manner. However, in addition to whether IL-1α interacts with the importin α family in mammalian cells, the actual functional involvement of importin α in the nuclear translocation of IL-1α was not examined in the mammalian or yeast cells in aforementioned reports.

The importin α family is also called karyopherin α, and there are seven subtypes in humans, which are classified into three subfamilies, α1, α2, and α3, based on the homology of the amino acid sequence (Table 1). There is approximately 50% homology between subfamilies, and within each subfamily, there is approximately 80% homology between subtypes except for importin α1 and importin α8. Each subtype is known to exhibit tissue-dependent expression (Table 1)^27^; furthermore these subtypes are expected to bind to intracellular substances and viruses in vivo in a substrate-specific manner^28^. In this study, we examined the interactions of all detectable importin α subtypes with IL-1α in a mammalian cell line (HeLa) and discussed the mechanism by which the importin α family participates in IL-1α nuclear translocation in mammalian cells.

**Table 1:** The subtypes of human importin α family.

Gene name	Protein name	Subfamily	Accession no.		Molecular weight (Da)	Tissue specificity	Function	References on interaction with IL-1α
			mRNA (NCBI)	Protein (UniProt)				
KPNA2	Importin subunit alpha-1	α2	NM_002266.4	P52292	57,862	Expressed ubiquitously	Functions in nuclear protein import as an adapter protein for nuclear receptor importin β1	N/A
KPNA4	Importin subunit alpha-3	α3	NM_002268.5	O00629	57,887	Highly expressed in testis, ovary, small intestine, heart, skeletal muscle, lung and pancreas, but barely detectable in kidney, thymus, colon and peripheral blood leukocytes		N/A
KPNA3	Importin subunit alpha-4	α3	NM_002267.4	O00505	57,811	Ubiquitous Highest levels in heart and skeletal muscle		N/A
KPNA1	Importin subunit alpha-5	α1	NM_002264.4	P52294	60,222	Expressed ubiquitously		N/A
KPNA5	Importin subunit alpha-6	α1	NM_002269.3	O15131	60,666	Testis		N/A
KPNA6	Importin subunit alpha-7	α1	NM_012316.5	O60684	60,030	Widely expressed		^26^
KPNA7	Importin subunit alpha-8	α2	NM_001145715.3	A9QM74	56,938	unknown	Functions in nuclear protein import	N/A

*N/A* not applicable.

## Results

### IL-1α expression and cNLS-dependent nuclear transport of IL-1α in HeLa cells

**Figure 1:**
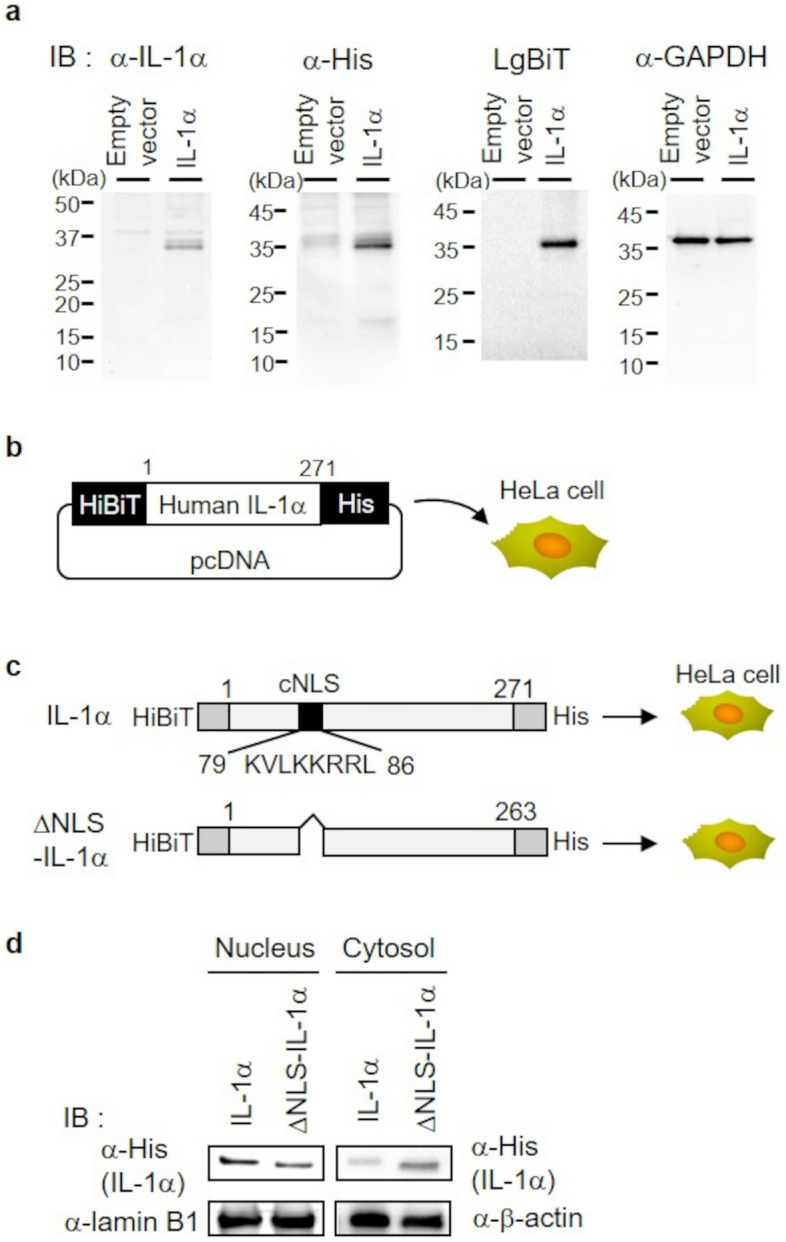
Construction of an expression system comprising HiBiT-tag- and His-tag fused IL-1α in HeLa cells and analysis of nuclear translocation of cNLS-deleted IL-1α. (**a**) At 24 h after transfection of HeLa cells with an empty vector or a vector incorporating N-terminal HiBiT-tag- and C-terminal His-tag fused IL-1α, the cells were collected and subjected to sodium dodecyl sulfate–polyacrylamide gel electrophoresis (SDS-PAGE), followed by immunoblotting with anti-IL-1α, anti-His, and anti-GAPDH antibodies or detection with LgBiT and its substrate. (**b**) Schematic of the *IL-1*α gene fused with a HiBiT-tag at the N-terminus and a His-tag at the C-terminus. pcDNA incorporating N-terminal HiBiT-tag and C-terminal His-tag-fused IL-1α plasmid was transiently expressed in HeLa cells. (**c**) Schematic representation of precursor IL-1α with the deleted cNLS sequence (KVLKKRRL). pcDNA incorporating N-terminal HiBiT-tag- and C-terminal His-tag fused IL-1α plasmid or a plasmid with deleted cNLS sequence from the full-length IL-1α plasmid were transiently expressed in HeLa cells. (**d**) Nuclear and cytoplasmic fractions of the HeLa cells expressing full-length IL-1α and cNLS-deleted IL-1α were subjected to immunoblotting with an anti-His, anti-lamin B1 and anti-β-actin antibodies. The full-length blots are shown in Supplementary Information, Fig. 1.

In this study, using HeLa cells expressing IL-1α, we examined the interactions between importin α family proteins and IL-1α. Since endogenous IL-1α may interfere with the interaction of expressed IL-1α and endogenous importin α, it is desirable to use cells with low endogenous IL-1α for sensitive detection of interactions. We performed Western blotting using lysate from HeLa cells and an anti-human IL-1α antibody. Consequently, immunoreactive bands were not detected for the precursor (33 kDa) or mature (C-terminal fragment: 17 kDa) fragments (lane of the empty vector in leftmost panel, Fig. 1a). This result showed that HeLa cells contain little or no endogenous IL-1α, making them suitable for this analysis. Then, Western blotting was performed using cell lysate obtained from HeLa cells that were transfected with human IL-1α fused with a HiBiT-tag at the N-terminus and a His-tag at the C-terminus (Fig. 1b). When detected with anti-IL-1α, anti-His antibodies, and HiBiT-tag luminescence, a distinct band of approximately 33 kDa was observed in each result, which is similar to the molecular weight of the IL-1α precursor (Fig. 1a). In contrast to the precursor IL-1α, the 17 kDa form of IL-1α was detected with low or no signal intensity. These findings are consistent with previous studies reporting that IL-1α mainly exists intracellularly as a precursor. Therefore, the IL-1α precursor was targeted for further analysis in this study.

Previously, immunofluorescence microscopy was utilized to explore the subcellular localization of GFP-fused IL-1α in HeLa cells; the findings demonstrated that GFP-fused IL-1α localizes to the nucleus in a cNLS-dependent manner^25,29^. The biochemical analysis in this study also revealed that IL-1α localization to the nucleus is cNLS-dependent. As shown in Fig. 1c, we prepared a construct of cNLS-deleted IL-1α fused with HiBiT, His tags (ΔNLS-IL-1α), and then transiently transfected it into HeLa cells. From the cell lysate, nuclear and cytoplasmic fractions were taken and put through Western blotting with an anti-His antibody. As a result, the signal intensity of IL-1α band in the nuclear fraction was reduced by the deletion of cNLS, whereas that of IL-1α band in the cytoplasmic fraction was enhanced by the deletion of cNLS (Fig. 1d). This suggests that deleting cNLS reduces IL-1α nuclear translocation. In general, molecules with a molecular weight of around ≤ 40 kDa may diffuse passively across the nuclear membrane pore^30^; accordingly, since the molecular weight of IL-1α is below this threshold, it is possible that the detection of IL-1α in the nuclear fraction, even in the absence of cNLS, is due to free diffusion of IL-1α into the nucleus. This biochemical approach could therefore be used to assess the cNLS-dependent nuclear localization of IL-1α. These findings supported the utility of this expression system for identifying the molecules involved in nuclear translocation.

### Analysis of interaction between IL-1α and importin α family proteins

To confirm whether the nuclear transport of IL-1α is mediated by the importin protein complex, we first analyzed the interaction of IL-1α with each importin α family protein. Initially, we performed a coimmunoprecipitation assay with an IL-1α-specific antibody. The importin α subtypes and antibody heavy chains have similar molecular weights (57–61 kDa) (Table 1); therefore, in addition to detecting importin α, the heavy chains of the antibody used for precipitation were detected nonspecifically. This masked the detection of importin α (results not shown). In order to provide clearer results, cobalt-based immobilized metal ion affinity chromatography (IMAC) was conducted on HeLa cells transfected with His-tag-fused IL-1α to isolate the His-tag fused protein complex in the current study.

**Figure 2:**
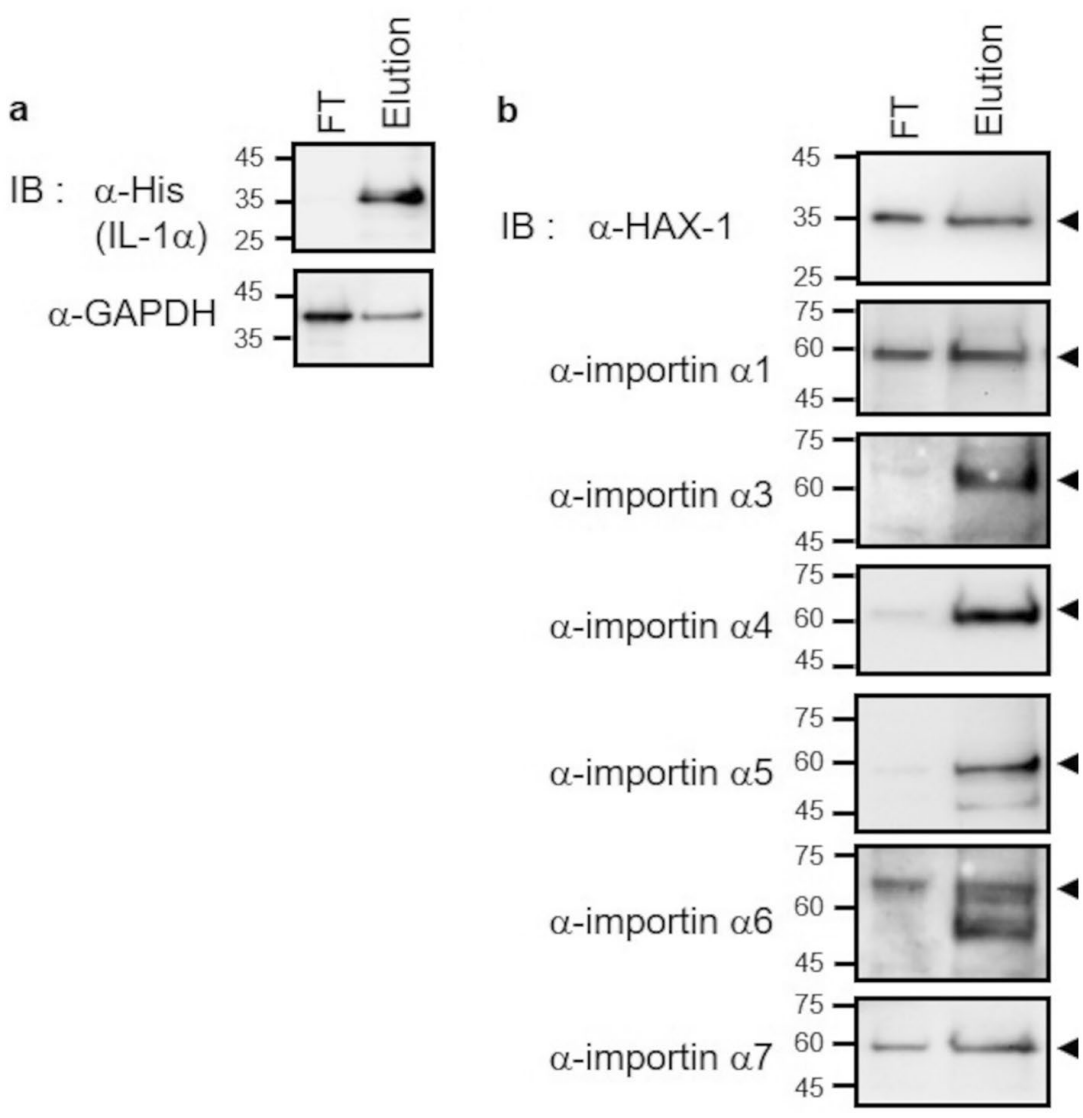
Identification of importin α subtypes interacting with IL-1α. The isolation of His-tag-fused IL-1α via IMAC was conducted using cell lysates from HeLa cells expressing His-tag-fused IL-1α. (**a**) Immunoblotting was performed using anti-His and anti-GAPDH antibodies; FT represents the flow-through fraction, and Elution represents the imidazole elution fraction. (**b**) anti-HAX-1, anti-importin α1, anti-importin α3, anti-importin α4, anti-importin α5, anti-importin α6, and anti-importin α7 antibodies were used for immunoblotting. Arrowheads indicate bands corresponding to the expected molecular weight of each protein. We confirmed the reproducibility of the results (Supplementary Fig. S1). The full-length blots are shown in Supplementary Information, Fig. 2.

To verify whether His-tag fused protein was isolated, the fractions obtained during IMAC were subjected to Western blotting using anti-His antibody, and a clear single band was detected corresponding to the molecular weight of IL-1α in the elution fraction expected to contain the His-tag fused protein (Fig. 2a). In contrast, no band was detected in the flow-through fraction (Fig. 2a). GAPDH (which does not interact with IL-1α) was detected with a stronger signal intensity in the flow-through fraction, whereas extremely low signal intensity was detected in the elution fraction (Fig. 2a). Moreover, HAX-1, which has been shown to interact with IL-1α in HEK293 cells^18^, was found in the elution fraction with a high signal intensity (Fig. 2b). These findings suggest that this assay can evaluate the interactions of the importin protein with IL-1α. To investigate the interaction of IL-1α with each importin α subtype using this assay, importin α1, α3, α4, α5, α6, and α7 in the flow-through and elution fractions were analyzed via Western blotting using each specific antibody (Supplementary Table 1). The results showed that for all importin α subtypes, a stronger signal was detected in the elution fraction than in the flow-through fraction (Fig. 2b). The anti-importin α8 antibody, however, was unable to detect importin α8 in HeLa cell lysates (data not shown). Thus, it is revealed that importin α1, α3, α4, α5, α6, and α7 interact with IL-1α in HeLa cells.

### Effect of importin α1 on the intracellular behavior of IL-1α

**Figure 3:**
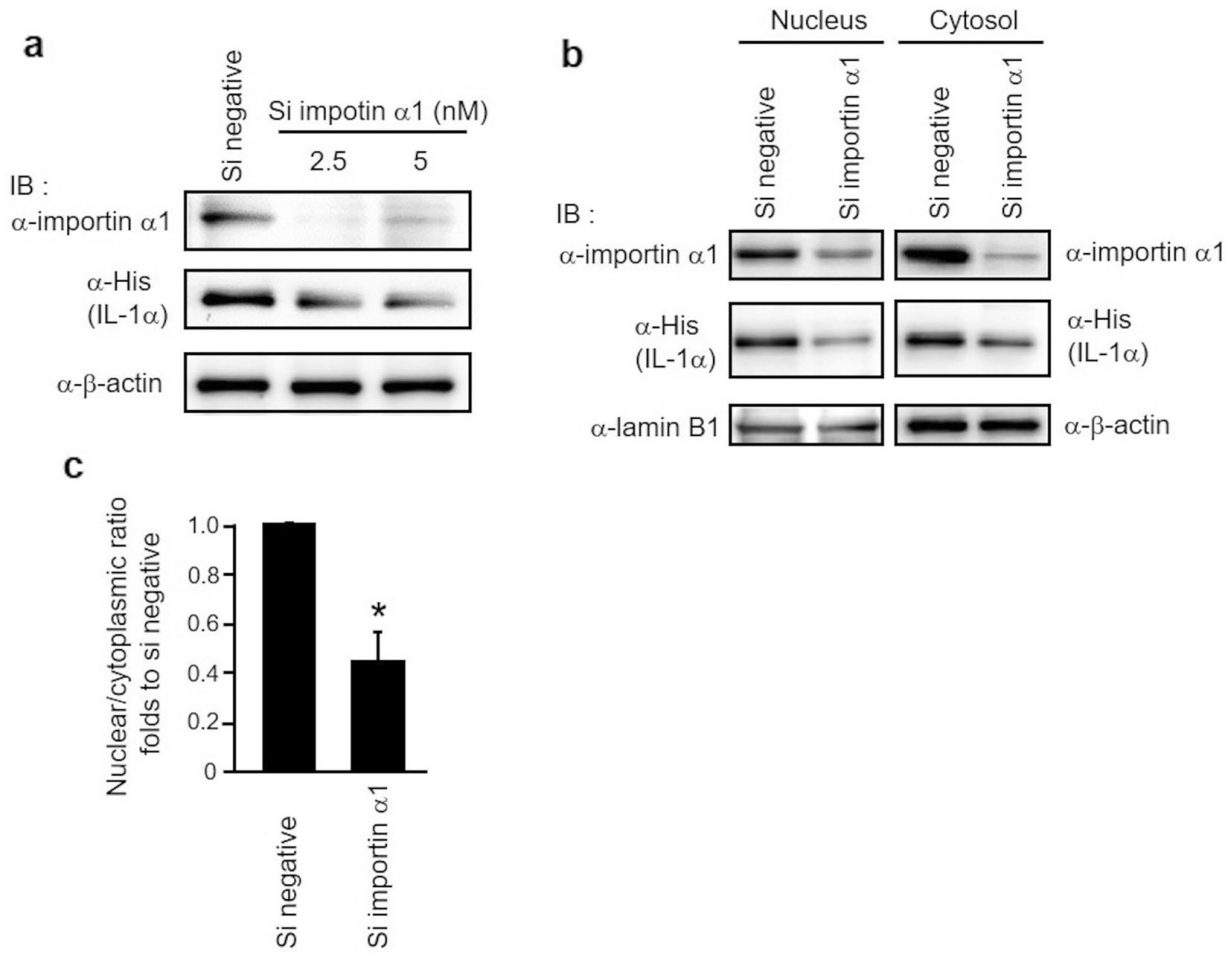
Analysis of the effect of importin α1 on IL-1α expression and nuclear translocation. (**a**) Cell lysates from HeLa cells transfected with 5 nM negative-control siRNA or 2.5 or 5 nM importin α1 siRNA and then transfected 24 h later with His-tag-fused IL-1α were subjected to SDS-PAGE and immunoblotting with anti-importin α1, anti-His, and anti-β-actin antibodies. (**b**) The nuclear and cytoplasmic fractions of HeLa cells transfected with importin α1 siRNA or negative-control siRNA and His-tag-fused IL-1α were obtained, and each fraction was then subjected to immunoblotting using anti-importin α1, anti-His, anti-lamin B1, and anti-β-actin antibodies. (**c**) IL-1α protein levels were measured in the nuclear and cytoplasmic fractions. Results are expressed as the nuclear/cytoplasmic ratio. Data are presented as the mean ± SD. Significant difference (P < 0.05) based on an unpaired Student’s *t*-test is indicated by asterisk (n ≥ 3). The full-length blots are shown in Supplementary Information, Fig. 3.

Among the importin α subtypes, the overexpression of importin α1 in various cancers, including breast, lung, esophageal, squamous cell, colon, prostate, and cervical cancers, has been reported in several studies^31–35^. Importin α1 expression is also higher in HeLa cells than in normal human cervical epithelial cells^36^; thus, importin α1 is considered to be functional in HeLa cells. Therefore, the present study focused on the effect of importin α1 on the nuclear translocation of IL-1α. Following that, we used different approaches to confirm the interaction between importin α1 and IL-1α, including coimmunoprecipitation of HeLa cells expressing His-tag-fused IL-1α and GST pulldown assay with GST-tag-fused importin α1 and His-tag-fused IL-1α; both approaches confirmed the interaction between importin α1 and IL-1α (Supplementary Figs. S2, S3). To examine the effect of importin α1 on the subcellular localization of IL-1α, we expressed His-tag fused IL-1α in HeLa cells transfected with siRNA targeting importin α1. The quantity of importin α1 protein was significantly reduced by siRNA transfection (Fig. 3a). Interestingly, in Western blotting using equal amounts of protein, IL-1α protein expression was also significantly decreased by importin α1 knockdown, whereas there was no difference in the expression of β-actin (Fig. 3a). To investigate the influence of importin α1 on IL-1α nuclear translocation, nuclear and cytoplasmic fractions were extracted from the cell lysate, and IL-1α was identified by Western blotting (Fig. 3b). In this analysis, although the protein amounts of the importin α1 siRNA-treated and negative siRNA-treated samples used were equal, the IL-1α signal intensities in the nuclear and cytoplasmic fractions of HeLa cells treated with importin α1 siRNA were lower than those in the nuclear and cytoplasmic fractions of HeLa cells treated with negative siRNA. By contrast, the signal intensities of lamin B1 in the nuclear fraction and β-actin in the cytoplasmic fraction were unchanged. This indicated that the knockdown of importin α1 can decrease the protein amount of IL-1α in HeLa cells. In addition, the nuclear/cytoplasmic ratio (i.e., the ratio of the amount of IL-1α protein in the nuclear fraction to that in the cytoplasmic fraction), was substantially reduced by knockdown of importin α1 (Fig. 3c). This indicated that importin α1 is related to the transport system of IL-1α in HeLa cells. To see whether IL-1α nuclear translocation was specifically regulated by importin α1, we investigated the effect of importin α4, which has been also shown to interact with IL-1α (Fig. 2b). The results showed that the knockdown of importin α4 had little effect on the protein amount and the nuclear translocation of IL-1α (Supplementary Fig. S4a,d). To further confirm the redundancy among importin α subtypes, we analyzed the effects of other importin α subtypes (importin α3, importin α5, and importin α7) on the nuclear transport of IL-1α. However, importin α6 was excluded from this analysis because it is expressed specifically in the testes. The results indicated that in addition to importin α1, knockdown of the gene expression of other importin α subtypes reduced the amount of IL-1α protein transported to the nucleus (Supplementary Fig. S5).

### Analysis of the behavior of importin β1 in IL-1α nuclear transport

**Figure 4:**
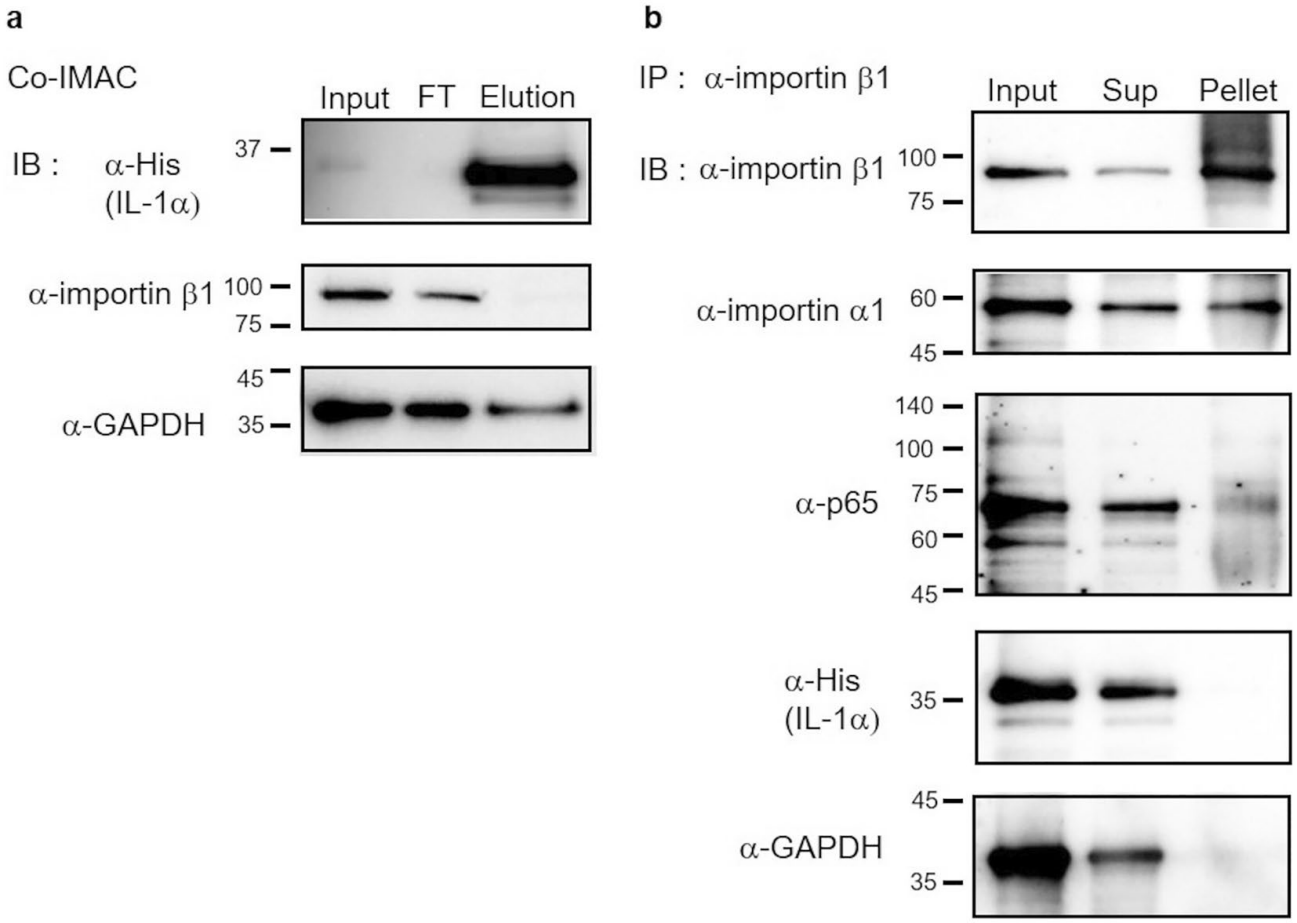
Analysis of the behavior of importin β1 in IL-1α nuclear translocation. (**a**) After isolation of His-tag-fused protein via IMAC from HeLa cells expressing His-tag-fused IL-1α, the obtained fractions during IMAC were analyzed using Western blotting with anti-His, anti-importin β1, and anti-GAPDH antibodies. *Input* lysate of HeLa cells before IMAC, *FT* flow-through fraction, *Elution* imidazole elution fraction. (**b**) Coimmunoprecipitation was performed with an anti-importin β1 antibody using HeLa cells expressing His-tag-fused IL-1α, and the input, supernatant fraction, and pellet fraction were analyzed by Western blotting using anti-importin β1, anti-importin α1, anti-p65, anti-His, and anti-GAPDH antibodies. *Input* lysate of HeLa cells before immunoprecipitation, *Sup* supernatant fraction, *Pellet* pellet fraction. The full-length blots are shown in Supplementary Information, Fig. 4.

To determine whether importin β1 was needed for nuclear transport of the IL-1α complex, we examined whether importin β1 was present in the complex. First, we examined the interaction between IL-1α and importin β1 by performing IMAC analysis using HeLa cells expressing His-tag fused IL-1α. The results showed that importin β1 was mainly detected in the flow-through fraction, which behaved similarly to GAPDH (Fig. 4a). Results suggested that importin β1 did not interact with the IL-1α complex. Importin β1 was further investigated using coimmunoprecipitation with an anti-importin β1 antibody. Consequently, importin β1 was detected in the pellet fraction, and so was importin α1. In addition, p65, which was transported to the nucleus by the importin α1/β1 complex, was coprecipitated with importin β1. However, IL-1α was detected in the supernatant fraction, exhibiting similar behavior as the negative control GAPDH (Fig. 4b). These findings demonstrated that importin β1 was not part of the IL-1α complex in HeLa cells. In addition, when importin β1 was knocked down using siRNA in HeLa cells expressing IL-1α, the nuclear translocation of IL-1α was not inhibited, reflecting non-interaction of importin β1 with IL-1α (Supplementary Fig. S4d). Furthermore, the nuclear translocation of IL-1α was analyzed in the presence of the importin β1 inhibitor importazole in HeLa cells expressing IL-1α. The nuclear translocation of p65, a substrate for importin β1 transport, was suppressed in the presence of 50 µM importazole, whereas IL-1α nuclear transport was unaffected (Supplementary Fig. S4e). These results indicated that importin β1 is not involved in the importin α-mediated nuclear transport of IL-1α.

## Discussion

Previously, Sahni et al. showed that IL-1α interacts with importin α7 using the Y2H screening assay^26^. However, the results were obtained from a comprehensive analysis opposed to an experiment focused solely on IL-1α, and it was possible that this interaction would not be replicated in mammalian cells. Therefore, it was necessary to verify the interaction between IL-1α and importin α subtypes in an experimental system using mammalian cells, which was not performed in the previous study. For the first time, we studied interactions between IL-1α and importin α subtypes in mammalian cells and discovered that IL-1α interacts with all detectable importin α subtypes, namely importin α1, α3, α4, α5, α6, and α7 (Fig. 2b). The results suggest that these subtypes are possibly involved in the nuclear translocation of IL-1α. However, the function of the importin α family in the nuclear translocation of IL-1α has not been investigated. Our analysis showed that reducing the expression of importin α1 suppressed IL-1α nuclear localization, demonstrating that among the importin α subtypes, at least importin α1 is involved in IL-1α nuclear translocation (Fig. 3b,c).

Redundancy in the importin α family was also examined because each importin α subtype interacted with IL-1α (Fig. 2b). We found that in addition to knockdown of importin α1, knockdown of most other importin α subtypes also reduced the amount of IL-1α protein transported to the nucleus (Supplementary Fig. S5b). This indicates that importin α subtypes are widely involved in the nuclear transport of IL-1α. However, the nuclear transport of IL-1α was unaffected by importin α4 knockdown (Supplementary Figs. S4d, S5b). These results suggest that importin α4 does not play a regulatory role in the nuclear transport of IL-1α. Although importin α4 might have a regulatory function in the nuclear transport of IL-1α, it is possible that its expression will be extremely low, in which case it will have a negligible effect on transport. To evaluate the substrate specificity of the importin α family, detailed analysis based on the absolute abundance of each importin α subtype is needed in the future. By contrast, Wan et al. detected multiple importin α subtypes in the same fraction in a proteomic analysis of isolated fractions obtained using various separation carriers, indicating that they interact with each another^37^. Furthermore, we also confirmed that the interaction between importin α1 and importin α3 using immunologic analysis (Supplementary Fig. S6). Thus, the elution of each importin α subtype together with IL-1α might reflect interactions between different importin α subtypes; namely, importin α1 and importin α3 might interact and function in a cooperative manner in vivo. Future studies on the interactions of importin α1 with subtypes other than importin α3 that considers the absolute abundance of each protein are needed. Furthermore, whether each importin α subtype binds directly or indirectly to IL-1α remains to be elucidated, and the contribution of the interactions between different importin α subtypes to the transport function of IL-1α requires additional study.

**Figure 5:**
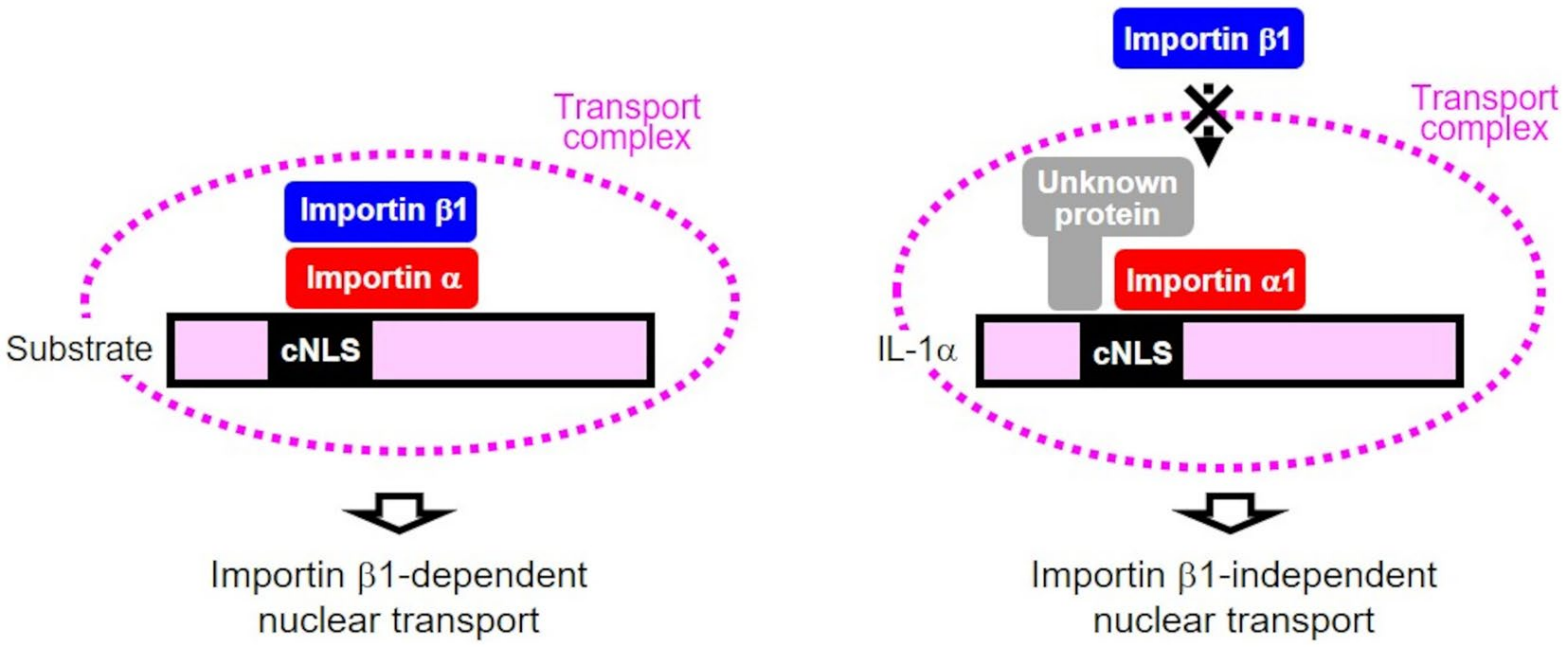
Components of complexes for nuclear translocation of IL-1α. Left side: general transport complex of substrate containing cNLS, which forms a complex with importin α, importin β1, and substrate transported to the nucleus in an importin β1-dependent manner. Right side: the IL-1α binds to importin α1 and an unknown protein, and is subsequently transported into the nucleus independently of importin β1.

In general, importin α binds to the cNLS of a cargo, and importin β1 binds to importin α, forming a complex that is transported into the nucleus in an importin β1-dependent manner (Fig. 5, left)^38–43^. The cNLS is usually found in the coils, loops, or intrinsically disordered regions so that its binding importin α can be facilitated. The NLS sequence of IL-1α (amino acids 79–86) is located in intrinsically disordered regions according to the AlphaFold model; therefore, this sequence can interact with importin α as a cNLS. In the present study, we demonstrated the following: (1) The cNLS deletion in IL-1α prevents the nuclear translocation of IL-1α (Fig. 1d); (2) importin α1 interacts with IL-1α, forming a complex (Fig. 2b); and (3) importin β1 is not included in the complex, suggesting that importin β1 does not involve IL-1α nuclear translocation (Fig. 4, Fig. S4d,e). These suggest that IL-1α translocates into the nucleus depending on importin α1 but independently of importin β1. In *Arabidopsis thaliana*, importin α binds with cNLS and mediates nuclear import independently of importin β1, but the mechanism is unclear^44^. What mechanism underlies the importin β1-independent nuclear translocation of IL-1α in HeLa cells? Importin α has an cNLS-binding domain as well as an importin β-binding domain (IBB) that binds to importin β1. The IBB domain also has cNLS-like properties, so both domains interact intramolecularly to prevent importin α from binding to importin β1^45–47^. After the cNLS of a cargo binds to the cNLS-binding domain of importin α, the IBB domain of importin α will become free to bind importin β1. In our findings, cNLS deletion significantly inhibited IL-1α nuclear translocation, suggesting that importin α1 binds to the cNLS of IL-1α, resulting in nuclear translocation despite the absence of importin β1 in the transport complex. This indicates that importin β1 cannot bind to importin α1, which is already attached to IL-1α’s cNLS. One suggested mechanism is that an unknown protein in the IL-1α nuclear transport complex inhibits the interaction between importin α1 and importin β1 (Fig. 5, right).

To further clarify whether HAX-1, which interacted with IL-1α (Fig. 2b), and its knockdown prevented IL-1α nuclear translocation (Supplementary Fig. S4d), is also involved in importin α1-mediated IL-1α translocation, the interaction between importin α1 and HAX-1 was examined by immunoprecipitation using anti-importin α1 and anti-HAX-1 antibodies. The results revealed no coprecipitation of importin α1 and HAX-1 (Supplementary Fig. S7). These results demonstrate that importin α1 and HAX-1 do not interact. Therefore, it was indicated that HAX-1 is involved in the nuclear translocation of IL-1α through a pathway independent of importin α1. Although HAX-1 is present in the nucleus^19,48–51^ and has been implicated in the nuclear transport of IL-1α by binding to the region containing the cNLS^19,52^, the specific transport mechanism is unclear, and further elucidation is required. It is possible that the unknown protein shown in Fig. 5, which presents a model of the regulation of the nuclear transport of IL-1α, plays a significant role in the nuclear transport of IL-1α mediated by importin α1. Conversely, Kotera et al. reported that importin α alone can transport Ca^2+^/calmodulin-dependent protein kinase type IV, which does not contain cNLS, to the nucleus^53^. The possibility of IL-1α nuclear translocation by importin α itself needs additional investigation.

This research indicated that importin α1 knockdown decreased the protein level of IL-1α in HeLa cells (Fig. 3). Recently, in addition to nuclear transport capacity, importin α1 is also noted for its various other functions^54,55^, such as the regulation of gene expression^56^, cell differentiation^57,58^, and spindle assembly^59^. In particular, the functions regarding protein polymerization and folding have been postulated: importin α/β inhibits the fibrillization of TDP-43, which is associated with amyotrophic lateral sclerosis and Alzheimer’s disease^60,61^, and in influenza A virus, importin α5 acts as a chaperone that inhibits the aggregation of nucleoprotein^62^. Hence, the interaction of importin α1 with IL-1α may also assist in the stabilization of IL-1α protein. The deletion of the cNLS in IL-1α lowered the quantity of IL-1α protein in the nucleus but had no impact on the overall quantity of IL-1α protein in the cell (Fig. 1d). Importin α1 therefore contributes to the regulation of IL-1α protein level by interacting with a domain other than the cNLS of IL-1α. This means that importin α1 interacts with IL-1α at multiple sites, similar to how HAX-1 interacts with IL-1α^18,19^. Ainscough et al*.*, reported that IL-1α was polyubiquitinated and exposed to proteasomal degradation in murine dendritic cells^63^. Although polyubiquitinated sites of IL-1α have not been identified, the cNLS sequence containing multiple lysine residues may correspond to polyubiquitinated sites, i.e., by masking the cNLS of IL-1α, importin α1 might be protecting IL-1α from proteasomal degradation. Importin α4 interacts with IL-1α (Fig. 2b), but knockdown did not lower IL-1α expression (Supplementary Fig. S4d). There can be substrate specificity between importin α subtypes for the regulation of IL-1α protein level. However, if the protein expression of importin α4 is extremely low, its effect on protein expression of IL-1α might not be reflected in the experimental results. To elucidate the mechanism underlying reduction of IL-1α protein expression via suppression of importin α1 expression and to discuss the substrate specificity of importin α family, a comprehensive analysis of the changes in protein abundance associated with the suppression of importin α1 expression need to be performed. In addition, targeted proteomic analysis should be conducted to determine the abundance of importin α subtypes and other related analyses would be needed.

To date, only HAX-1 has been identified as a molecule that directly regulates IL-1α nuclear translocation, and the mechanism of the importin α dependent nuclear translocation of IL-1α with cNLS has not been directly analyzed. This research showed that all detected importin α subtypes interact with IL-1α in HeLa cells. Among these subtypes, we discovered that at least importin α1-mediated nuclear translocation of IL-1α occurs and that the transport pathway is independent of importin β1. Moreover, importin α1 is involved in the regulation of the IL-1α protein level in HeLa cells. To clarify the mechanism by which the IL-1α-importin α1 complex is translocated to the nucleus, detailed and careful analyses, including proteomic analysis, are required to identify the proteins that interact with the complex during the nuclear transport of IL-1α in a spatiotemporal manner. Our results imply that importin α1 may be valuable as a potential therapeutic target for all IL-1α-related diseases, whether intracellular and extracellular.

## Materials and methods

### Reagents

Monoclonal mouse anti-IL-1α (sc-271618), anti-GAPDH (sc-47724), anti-importin α1 (sc-55538), anti-importin α4 (sc-514101), anti-importin α5 (sc-101292), anti-importin α7 (sc-390055) antibodies were purchased from Santa Cruz Biotechnology (Dallas, TX, USA). Proteintech (Rosemont, IL, USA) provided polyclonal rabbit anti-β-actin (20536-1-AP) and anti-lamin B1 (12987-1-AP) antibodies, as well as monoclonal mouse anti-GST tag antibody (66001-2-Ig). Monoclonal mouse anti-His antibody (D291-3) was purchased from MBL (Tokyo, Japan). Monoclonal mouse anti-importin α3 (ab53751) and anti-importin β1 antibodies (ab2811) were purchased from Abcam (Cambridge, UK). Monoclonal mouse anti-importin α6 antibody (H00003841-M01) was purchased from Abnova (Taipei, Taiwan). Monoclonal rabbit anti-p65 antibody (8242) was purchased from Cell Signaling Technology (Danvers, MA, USA). For more information on anti-importin antibodies, see Supplementary Table 1. Verification of the specificity of the antibody against each importin α subtype is shown in Supplementary Fig. S8.

### Cell culture and maintenance

HeLa cells were cultured in Dulbecco’s Modified Eagle’s Medium supplemented with 10% fetal calf serum, 50 μg/mL streptomycin, and 50 U/mL penicillin (Sigma-Aldrich, St. Louis, MO) for 3 days in a humidified incubator (5% CO_2_, 37 °C). The cells were maintained by passage every 2–3 days.

### Construction of plasmid vectors

Using the Quick-Change site-directed mutagenesis kit (Agilent, Böblingen, Germany), expression vector (HiBiT-IL-1α-His) containing the N-terminal HiBiT-tag (11 amino acids [VSGWRLFKKIS]) and the C-terminal His-tag was constructed, and pcDNA-IL-1α vector was used as a template^64^. The cNLS-deletion mutant of IL-1α (∆NLS) was constructed using the HiBiT-IL-1α-His vector as a template and the above-mentioned kit. Plasmids were sequenced to ensure that no undesired mutations were present.

### Transfection experiment

HeLa cells were seeded in a 6-well plate (5 × 10^5^ cells/well) 1 day before transfection with the expression vector. The expression vector was transfected according to the manufacturer’s instructions using Lipofectamine 3000 reagent (Thermo Fisher Scientific, Waltham, MA). After incubation for 24 h, the transfected cells were collected and analyzed. For RNA interference, 2.5 and 5 nM siRNA targeting importin α1 (Silencer select ID s7922, Thermo Fisher Scientific), 5 nM siRNA targeting importin α4 (Silencer select ID s7923, Thermo Fisher Scientific), HAX-1 (Silencer select ID s20458, Thermo Fisher Scientific), importin β1 (Silencer select ID s7917, Thermo Fisher Scientific), and 5 nM universal negativecontrol siRNA (Silencer select ID 4390843, Thermo Fisher Scientific) were transfected using Lipofectamine RNAiMAX (Thermo Fisher Scientific) for 24 h before transfecting the cells with the expression vector, as per the manufacturer’s instructions.

### Protein–protein interaction analysis

To purify the His-tagged protein and its binding proteins, IMAC was performed using Dynabeads His-Tag Isolation and Pulldown kit (Thermo Fisher Scientific). Cobalt was utilized as a tetradentate metal chelator in this strategy to bind to His-tagged proteins. In brief, HeLa cells transfected with His-tagged IL-1α were lysed in 700 μL of cell lysis buffer and then treated with 50 μL of cobalt-coated magnetic beads for 5 min at room temperature. The solutions were then placed on a magnet for 2 min to wash the magnetic beads 4 times with buffer containing 50 mM Na-phosphate (pH 8.0), 300 mM NaCl, and 0.01% Tween 20. The flow-through obtained from the first wash was used for Western blotting analysis as a fraction containing molecules that did not bind to the His-tag fused protein. The His-tag fused protein and its binding proteins were extracted from magnetic beads using 100 μL of His-elution buffer (300 mM imidazole, 50 mM sodium phosphate (pH 8.0), 300 mM NaCl, and 0.01% tween 20). This process was performed for 15 min at room temperature. The samples (the flow-through fraction obtained from the first wash, referred to as “FT”, and the fraction obtained from His-elution, referred to as “Elution”) were then used for Western blotting analysis. The amount of magnetic beads was adjusted according to the amount of protein present in the sample.

To determine whether importin β1 interacts with IL-1α complex, we utilized Dynabeads Coimmunoprecipitation Kit (Thermo Fisher Scientific) and followed the manufacturer’s instructions for immunoprecipitation. The lysis buffer supplied with the kit was used to lyse the cells. For antibody immobilization, anti-importin β1 antibody (Abcam ab2811) was linked to magnetic beads. First, anti-importin β1 antibody was coupled to Dynabeads M-270 Epoxy at 37 °C for overnight. The cell lysate and antibody-coated Dynabeads M-270 Epoxy were incubated for 30 min at 4 °C. The tube containing the sample was then placed on a magnet to collect the beads, and the supernatant was removed. This step was repeated to wash the beads. Extraction buffer from the kit was used to collect the target protein complexes. Thereafter, the samples (the supernatant fraction obtained from the first wash, referred to as “Sup” and the fraction obtained from elution buffer, referred to as “Pellet”) were used for Western blotting. Alternatively, to confirm the interaction between importin α1 and IL-1α, HeLa cells transfected with empty vector or His-tag fused *IL-1*α gene were utilized for coimmunoprecipitation with anti-His antibody. The precipitates were collected using Protein G Sepharose™ 4 fast flow (GE Healthcare Bioscience, Piscataway, NJ) after incubating the cell lysate with anti-His antibody and were subjected to Western blotting analysis.

HeLa cells coexpressing His-tag-fused IL-1α and GST-fused importin α1 were utilized for GST pulldown assays. Glutathione SepharoseTM 4 Rapid Flow (GE Healthcare Bioscience) was utilized to capture GST-fused proteins. Western blotting using an anti-His antibody was used to detect IL-1α in GST-fusion protein complexes. Additional procedures included collecting His-tag fused proteins on Ni–NTA agarose beads (Qiagen, Hilden, Germany) and blotting the samples with an anti-GST antibody.

### Western blotting experiment

HeLa cells transfected with the expression vector were washed twice with cold PBS before being lysed with cell lysis buffer (1% Triton X-100/10 mM Tris–HCl buffer [pH 8.0]). Cytoplasmic and nuclear extracts were obtained from the cell lysate using NE-PER™ nuclear and cytoplasmic extraction kit (Thermo Fisher Scientific). Protein concentrations were measured using the Bio-Rad protein assay (Bio-Rad Laboratories, Inc., Hercules, CA), and then Western blotting was performed. Briefly, protein mixtures were resolved by SDS-PAGE under reducing conditions of 8% or 12% gels, which were then electrotransferred onto PVDF membranes. After blocking nonspecific binding using PBST containing 1% BSA, each blot was incubated overnight with the primary antibody at 4 °C and subsequently with the HRP-conjugated secondary antibody for 1 h at room temperature. Supplementary Table 1 describes the primary antibodies against importin α and importin β1. The blots were visualized with ECL Prime Western Blotting Detection Reagent (Cytiva, Tokyo, Japan). Super Signal Ultra Chemiluminescent Substrate (Thermo Fisher Scientific) was used to detect importins α3 and α6. To demonstrate equivalent protein loading, GAPDH or β-actin levels in whole-cell lysates, β-actin levels in the cytoplasmic fraction, and lamin B1 levels in the nuclear fraction were evaluated. The resulting bands were analyzed using the iBright imaging system (Thermo Fisher Scientific, Waltham, MA, USA).

### Statistical analysis

Data are expressed as mean ± SD. Differences between two groups were assessed using an unpaired two-tailed Student’s t-test. Data were analyzed using the iBright imaging system. The results were considered significant at P < 0.05.

### Supplementary Information

Supplementary Figures.

Supplementary Information.

Supplementary Table 1.

## Data Availability

The datasets used in the current study are available from the corresponding author on reasonable request.

## Abbreviations

cNLS: Classical nuclear localization signal
HAX-1: HCLS1-associated protein X-1
IL-1α: Interleukin 1 alpha
IL-1R1: IL-1 receptor type 1
IMAC: Immobilized metal affinity chromatography
Importin α: Importin subunit alpha
Importin β: Importin subunit beta
SDS-PAGE: Sodium dodecylsulfate-polyacrylamide gel electrophoresis
Y2H: Yeast two-hybrid
