# Supplementary material for: Analysis of the effects of importin α1 on the nuclear translocation of IL-1α in HeLa cells
Source: Sci Rep. 2024 Jan 15;14:1322. doi: 10.1038/s41598-024-51521-w (PMC10789739; doi:10.1038/s41598-024-51521-w)
Supplement: Supplementary file 1 — Supplementary Figures. [file 41598_2024_51521_MOESM1_ESM.pdf]

Analysis of the effects of importin α1 on the nuclear translocation of IL-1α in HeLa cells

Akiko Yamada,[1,2] *† Kiyotaka Wake,[3,4]* Saya Imaoka,[1,2] Mitsuru Motoyoshi,[3,5] Takenori Yamamoto,[6,7] Masatake Asano[1,2]

[1]Department of Pathology, Nihon University School of Dentistry, 1-8-13, Kanda-Surugadai, Chiyoda-ku, Tokyo 101-8310, Japan

[2]Division of Immunology and Pathobiology, Dental Research Center, Nihon University School of Dentistry, 1-8-13, Kanda-Surugadai, Chiyoda-ku, Tokyo 101-8310, Japan

[3]Department of Orthodontics, Nihon University School of Dentistry, 1-8-13, Kanda-Surugadai, Chiyoda-ku, Tokyo 101-8310, Japan

[4]Division of Oral Structural and Functional Biology, Nihon University Graduate School of Dentistry, 1-8-13, Kanda-Surugadai, Chiyoda-ku, Tokyo 101-8310, Japan

[5]Division of Clinical Research, Dental Research Center, Nihon University School of Dentistry, 1-8-13, Kanda-Surugadai, Chiyoda-ku, Tokyo 101-8310, Japan

[6]Division of Molecular Target and Gene Therapy Products, National Institute of Health Sciences, 3-25-26, Tonomachi, Kawasaki-ku, Kawasaki-shi, Kanagawa 210-9501, Japan

[7]Institute for Genome Research, Tokushima University, Kuramotocho-3, Tokushima 770-8503, Japan

Supplementary figure 1

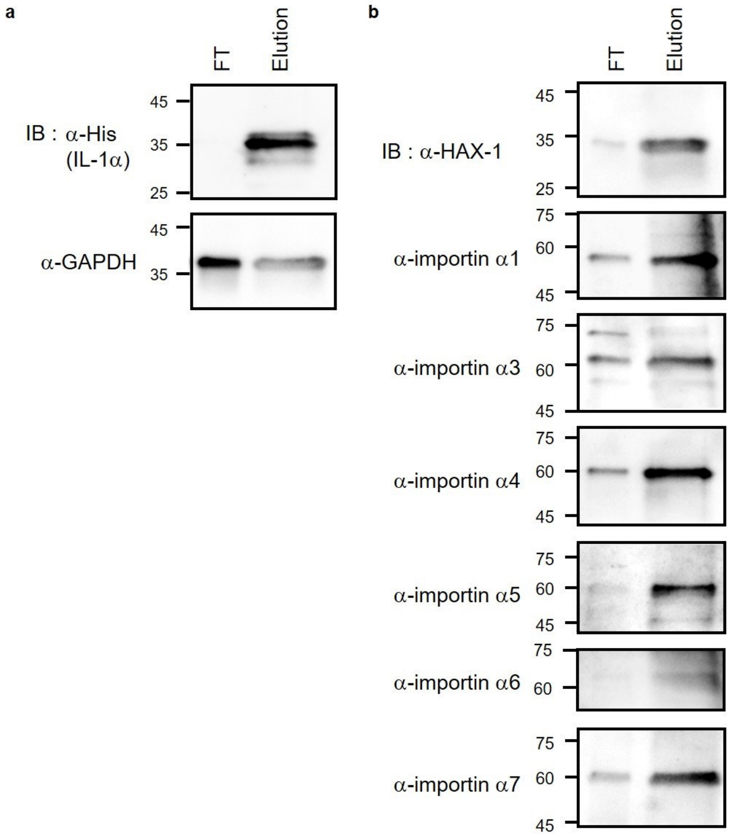

Supplementary Figure S1. Identification of importin α subtypes that interact with IL-1α. His-tag-fused IL-1α was isolated via IMAC from cell lysates obtained from HeLa cells expressing His-tag-fused IL-1α. (**a**) Immunoblotting was performed using anti-His and anti-GAPDH antibodies; FT represents the flow-through fraction, and Elution represents the imidazole elution fraction. (**b**) Anti-HAX-1, anti-importin α1, anti-importin α3, anti-importin α4, anti-importin α5, anti-importin α6, and anti-importin α7 antibodies were used for immunoblotting. The full-length blots are shown in Supplementary Information, Fig. S1.

Supplementary figure 2

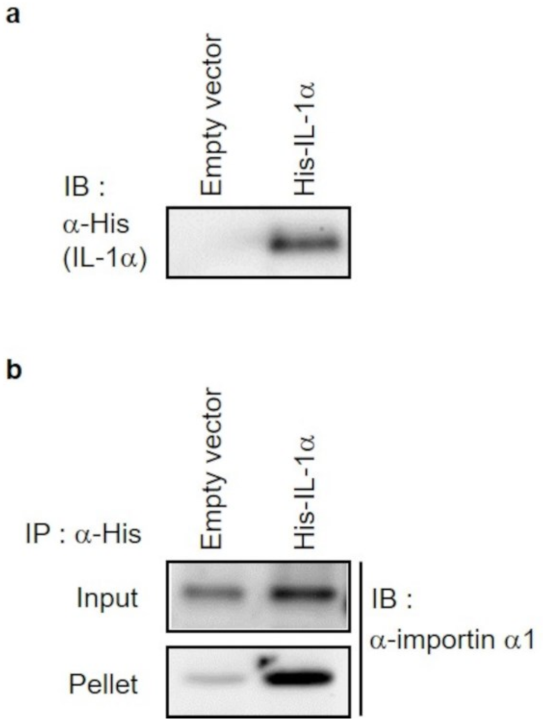

Supplementary Figure S2. Analysis of the interaction between importin α1 and IL-1α during coimmunoprecipitation. (**a**) Expression of His-tag-fused *IL-1α* gene in HeLa cells was confirmed by Western blotting using anti-His antibody. (**b**) Coimmunoprecipitation of HeLa cells shown in (**a**) was performed using anti-His antibody. The obtained fractions (input; lysate of HeLa cells before immunoprecipitation, and pellet; pellet fraction) were subjected to Western blotting to detect importin α1. The signal intensity of importin α1 in the input sample in IL-1α-expressing HeLa cells was approximately equivalent to that in the empty vector-expressing cells, but in the pellet fraction, the signal intensity of importin α1 was significantly stronger in IL-1α-expressing HeLa cells than in empty vector-expressing cells. Importin α1, which interacts with IL-1α, was enriched in the pellet fraction. The full-length blots are shown in Supplementary Information, Fig. S2.

Supplementary figure 3

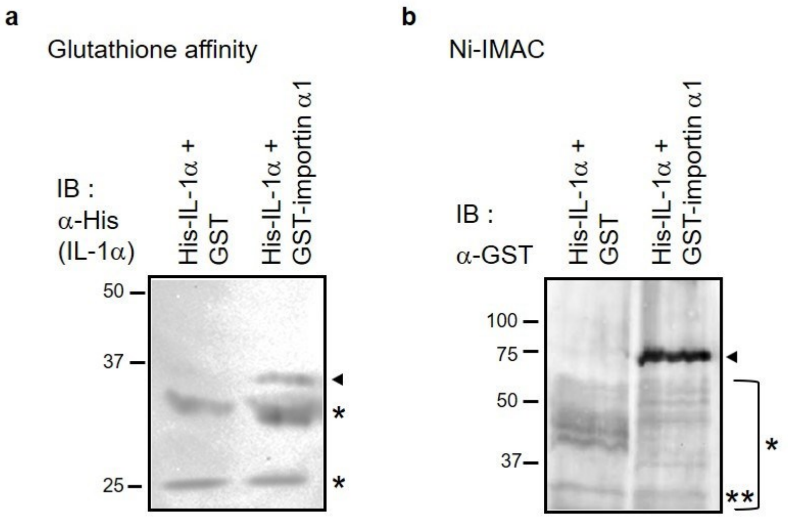

Supplementary Figure S3. Analysis of the interaction between importin α1 and IL-1α by GST pulldown assay. GST-importin α1 and His-IL-1α coexpressing HeLa cells were used for the analysis. (**a**) GST-fusion protein complexes were purified using a glutathione affinity matrix, and IL-1α was detected using an anti-His antibody in the collected complexes. Since the signal intensities of the non-specific bands were nearly equivalent between the two lanes ("His-IL-1α+GST" and "His-IL-1α+GST-importin α1"), it was assumed that almost equal amounts of protein were blotted. (**b**) His-tag fusion protein complexes were collected using Ni-IMAC, and importin α1 was identified in the collected complexes using an anti-GST antibody. Since the signal intensities of non-specific bands located at the same molecular weight in the "His-IL-1α+GST" and "His-IL-1α+GST-importin α1" lanes (indicated by double asterisks) were nearly equivalent, these lanes contained approximately equal amounts of protein. Arrowheads indicate bands corresponding to the expected molecular weight of each protein. Asterisks indicate non-specific bands. The full-length blots are shown in Supplementary Information, Fig. S3.

Supplementary figure 4

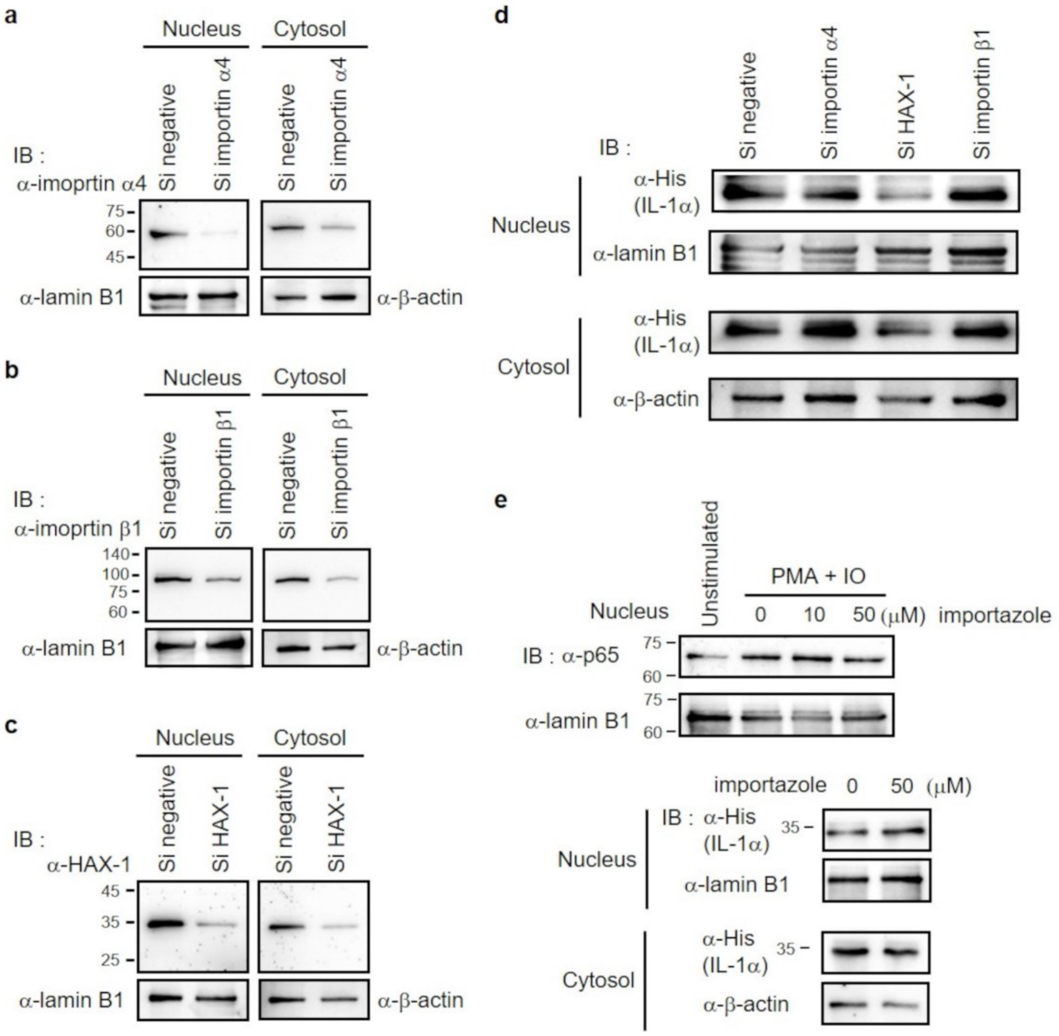

Supplementary Figure S4. Analysis of the effects of importin α4, importin β1, and HAX-1 on the nuclear translocation of IL-1α. (**a**), (**b**), (**c**) The cell lysates of HeLa cells transfected with siRNA targeting importin α4, importin β1, and HAX-1, respectively, and transfected with His-tag-fused IL-1α after 24 h were subjected to Western blotting with anti-importin α4, anti-importin β1, anti-HAX-1, anti-lamin B1, and anti-β-actin antibodies. (**d**) HeLa cells used in (**a**), (**b**), and (**c**) were fractionated into nuclear and cytoplasmic fractions, and Western blotting was performed for each fraction with anti-IL-1α, anti-lamin B1, and anti-β-actin antibodies. (**e**) Nuclear fractions were extracted from HeLa cells stimulated with 20 ng/ml PMA and 1 μM ionomycin for 3 h in the presence of 10 μM or 50 μM importazole and subjected to Western blotting with anti-p65 and anti-lamin B1 antibodies. HeLa cells expressing IL-1α were also treated with 50 μM importazole for 3 h, and the nuclear and cytoplasmic fractions were subjected to Western blotting using anti-His, anti-lamin B1 and anti-β-actin antibodies. PMA, phorbol-12-myristate-13-acetate; IO, ionomycin. The full-length blots are shown in Supplementary Information, Fig. S4.

Supplementary figure 5

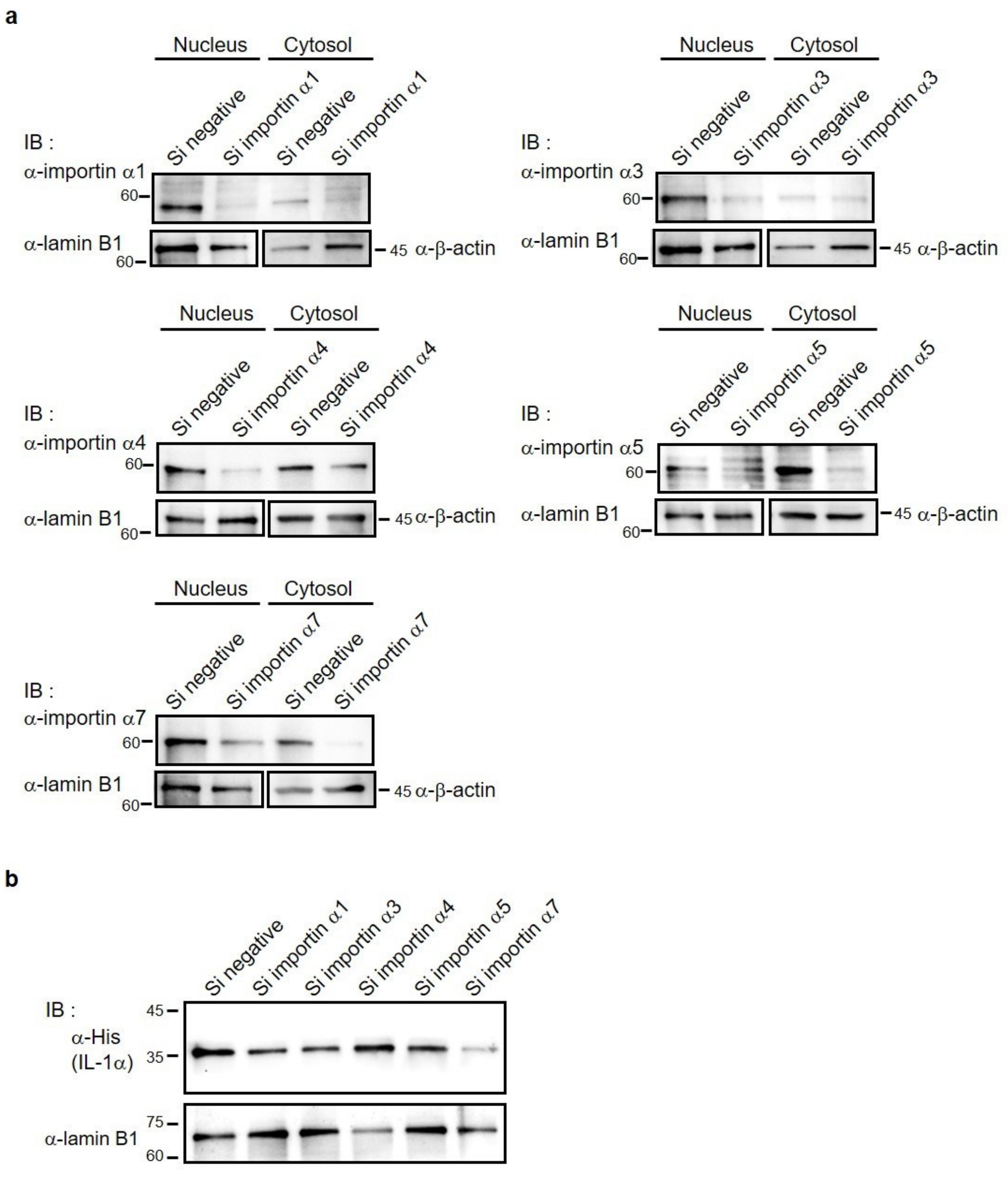

Supplementary Figure S5. Analysis of the effect of each importin α subtype on the nuclear translocation of IL-1α. The nuclear proteins were extracted from HeLa cells transfected with siRNA targeting importin α1, importin α3, importin α4, importin α5, and importin α7 together with His-tag–fused IL-1α, and Western blotting was performed using specific antibodies against each importin α subtype (**a**) or anti-His and anti-lamin B1 antibodies (**b**). The full-length blots are shown in Supplementary Information, Fig. S5.

Supplementary figure 6

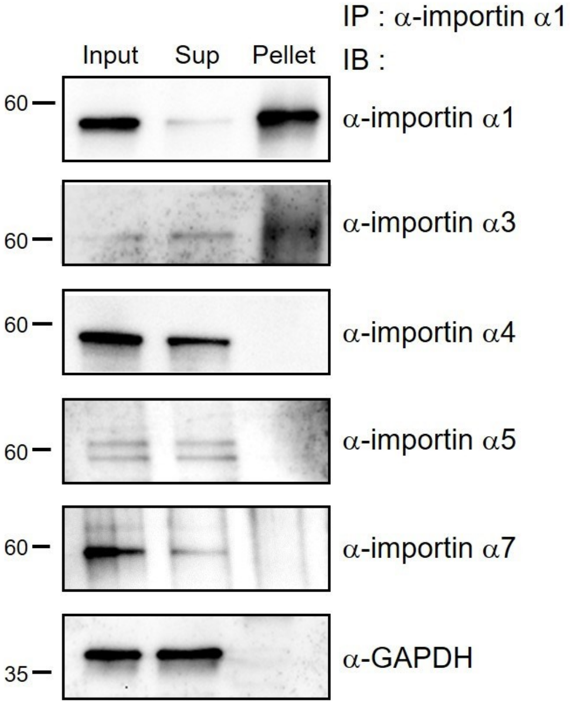

Supplementary Figure S6. Coimmunoprecipitation of HeLa cell lysates was performed using importin α1 antibody, and the input, supernatant fraction, and pellet fraction were subjected to Western blotting using antibodies specific for each importin α subtype. GAPDH, which does not interact with importin α1, was used as a negative control. Input, lysate of HeLa cells before immunoprecipitation; Sup, supernatant fraction; Pellet, pellet fraction. The full-length blots are shown in Supplementary Information, Fig. S6.

Supplementary figure 7

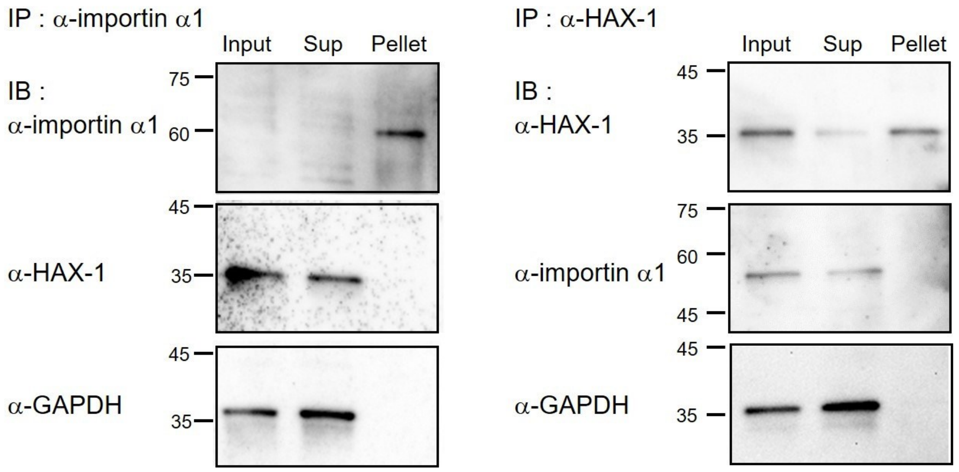

Supplementary Figure S7. Coimmunoprecipitation was performed with an anti-importin α1 antibody (left panel) or an anti-HAX-1 antibody (right panel) using HeLa cells expressing His-tag–fused IL-1α. The input, supernatant fraction, and pellet fraction were analyzed by Western blotting with anti-importin α1, anti-HAX-1, and anti-GAPDH antibodies. Input, lysate of HeLa cells before immunoprecipitation; Sup, supernatant fraction; Pellet, pellet fraction. The full-length blots are shown in Supplementary Information, Fig. S7.

Supplementary figure 8

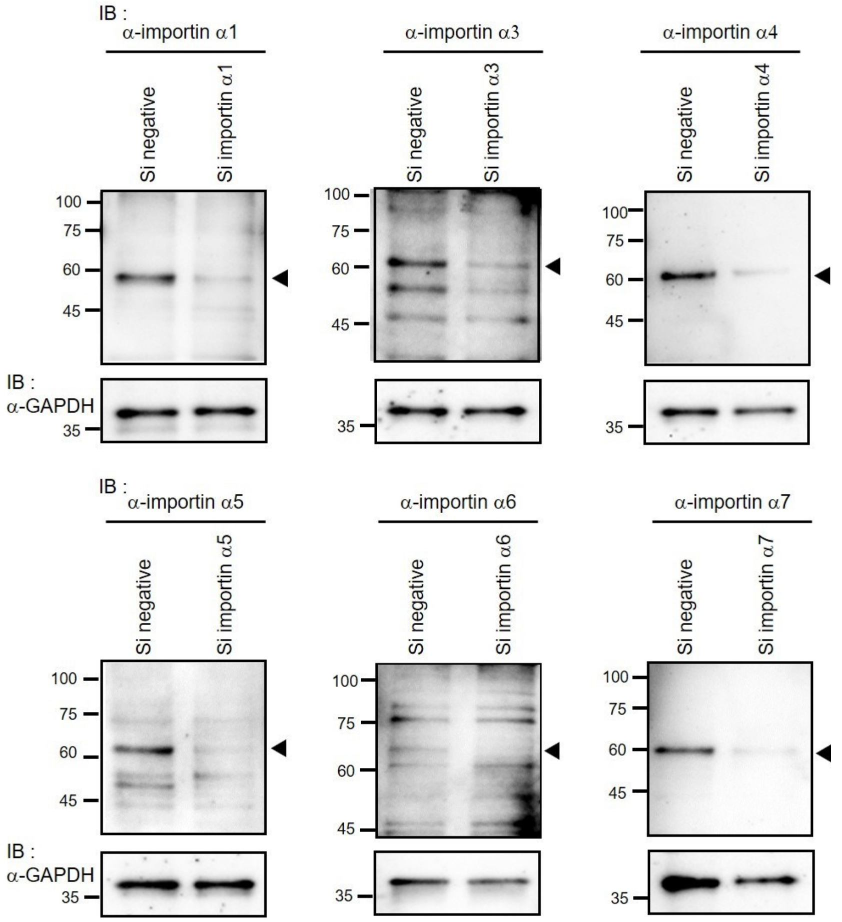

Supplementary Figure S8. Verification of the specificity of the antibodies used to detect different importin α subtypes. HeLa cells were transfected with siRNA targeting each importin α subtype, including importin α1 (Silencer select ID s7922, Thermo Fisher Scientific), importin α3 (Silencer select ID s7926, Thermo Fisher Scientific), importin α4 (Silencer select ID s7923, Thermo Fisher Scientific), importin α5 (Silencer select ID s223979, Thermo Fisher Scientific), importin α6 (Silencer select ID s7929, Thermo Fisher Scientific), and importin α7 (Silencer select ID s24241, Thermo Fisher Scientific). After 24-h transfection, cell lysates were subjected to SDS-PAGE, and Western blotting was performed using specific antibody. The results indicated that for all importin α subtypes, the target bands detected in cells transfected with negative control siRNA were undetectable following transfection with specific siRNAs. This indicated that each antibody specifically detected the indicated importin α subtype. The full-length blots are shown in Supplementary Information, Fig. S8.
