# Supplementary material for: Analysis of the effects of importin α1 on the nuclear translocation of IL-1α in HeLa cells
Source: Sci Rep. 2024 Jan 15;14:1322. doi: 10.1038/s41598-024-51521-w (PMC10789739; doi:10.1038/s41598-024-51521-w)

Figure 1

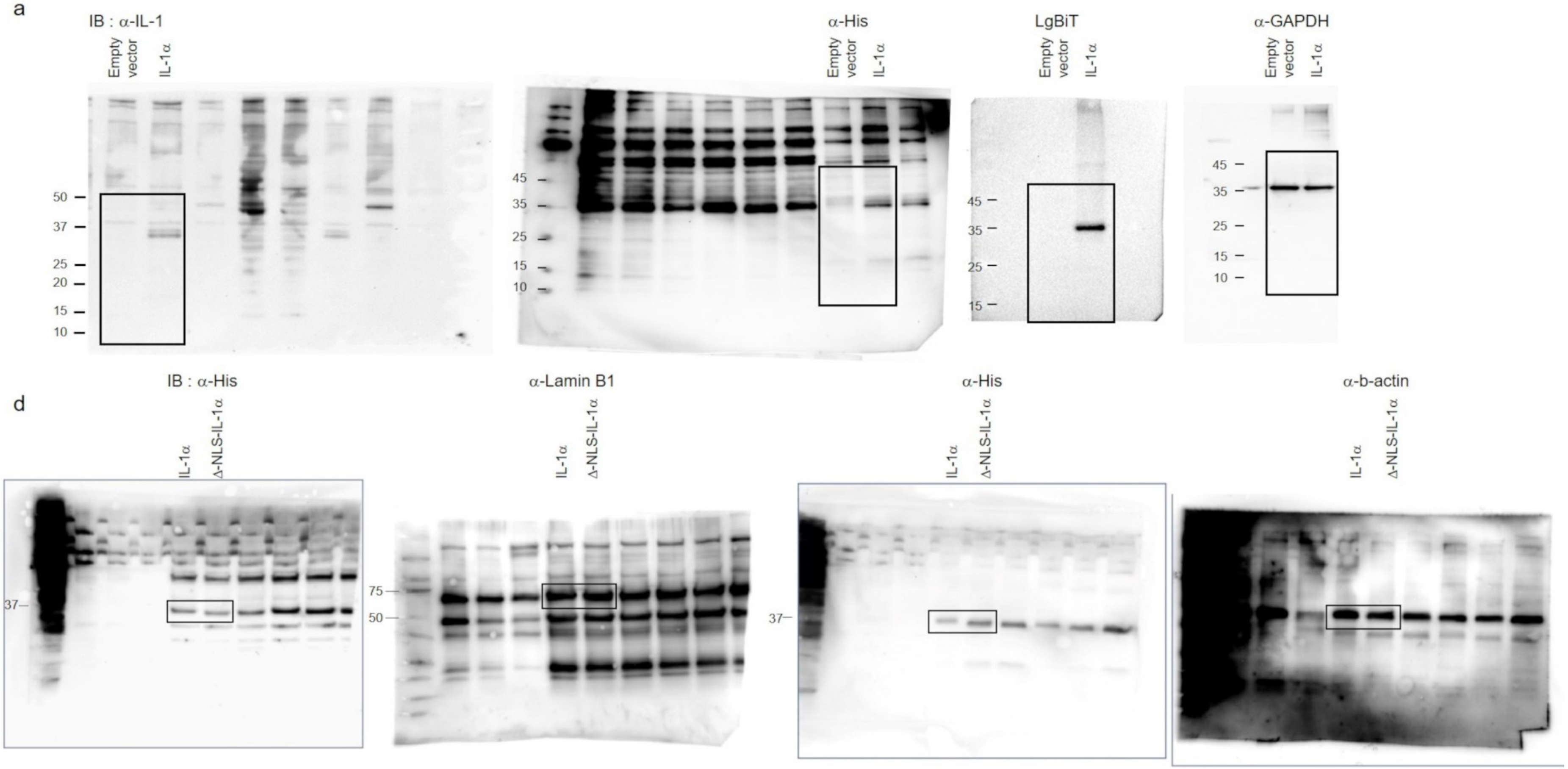

a
IB : α-IL-1
Empty vector
IL-1α
50
37
25
20
15
10
α-His
Empty vector
IL-1α
45
35
25
15
10
LgBiT
Empty vector
IL-1α
45
35
25
15
α-GAPDH
Empty vector
IL-1α
45
35
25
15
10
d
IB : α-His
IL-1α
Δ-NLS-IL-1α
37
α-Lamin B1
IL-1α
Δ-NLS-IL-1α
75
50
α-His
IL-1α
Δ-NLS-IL-1α
37
α-b-actin
IL-1α
Δ-NLS-IL-1α

Figure 2

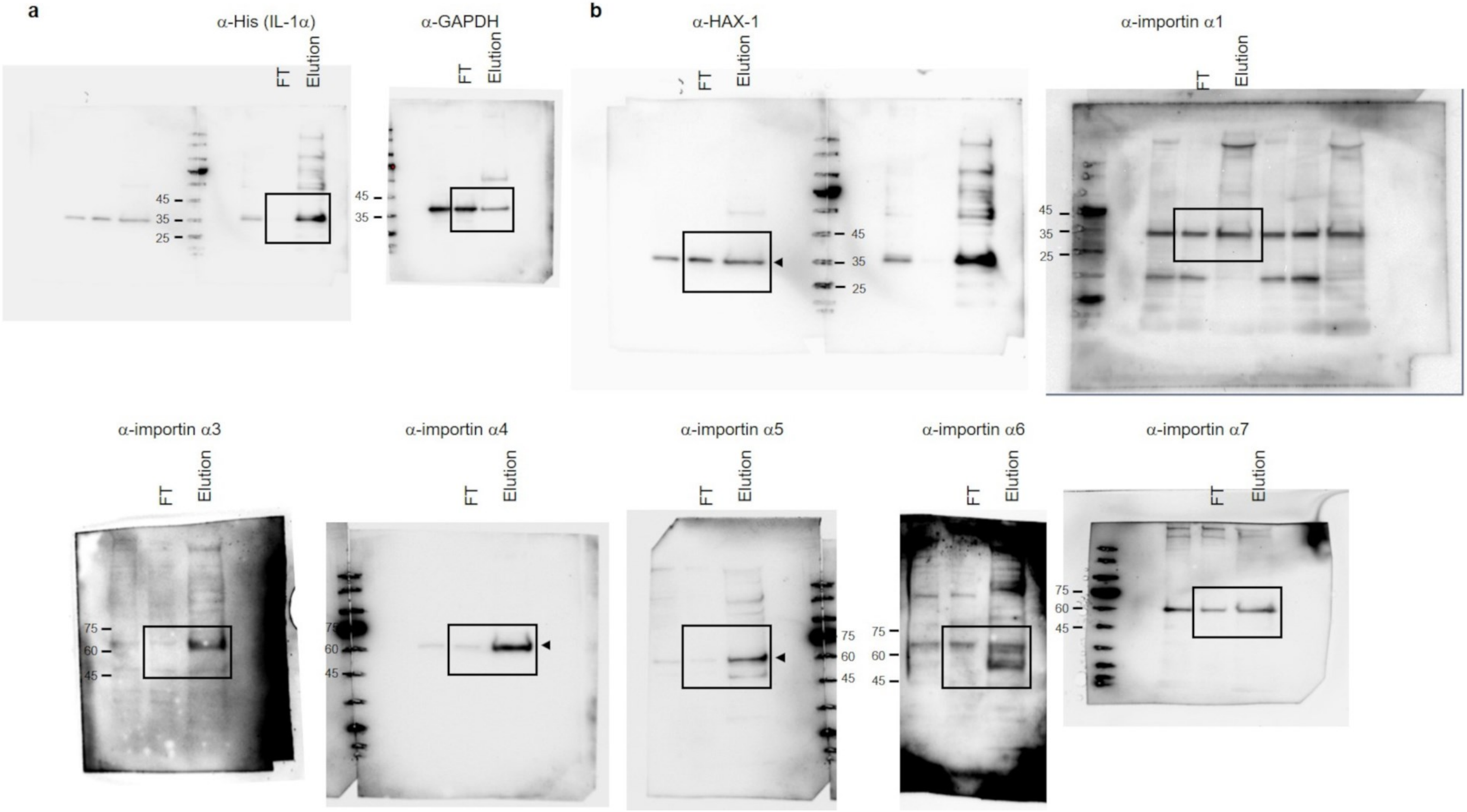
a
α-His (IL-1α)
FT
Elution
45
35
25
α-GAPDH
FT
Elution
45
35
b
α-HAX-1
FT
Elution
45
35
25
α-importin α1
FT
Elution
45
35
25
α-importin α3
FT
Elution
75
60
45
α-importin α4
FT
Elution
75
60
45
α-importin α5
FT
Elution
75
60
45
α-importin α6
FT
Elution
75
60
45
α-importin α7
FT
Elution
75
60
45

Figure 3

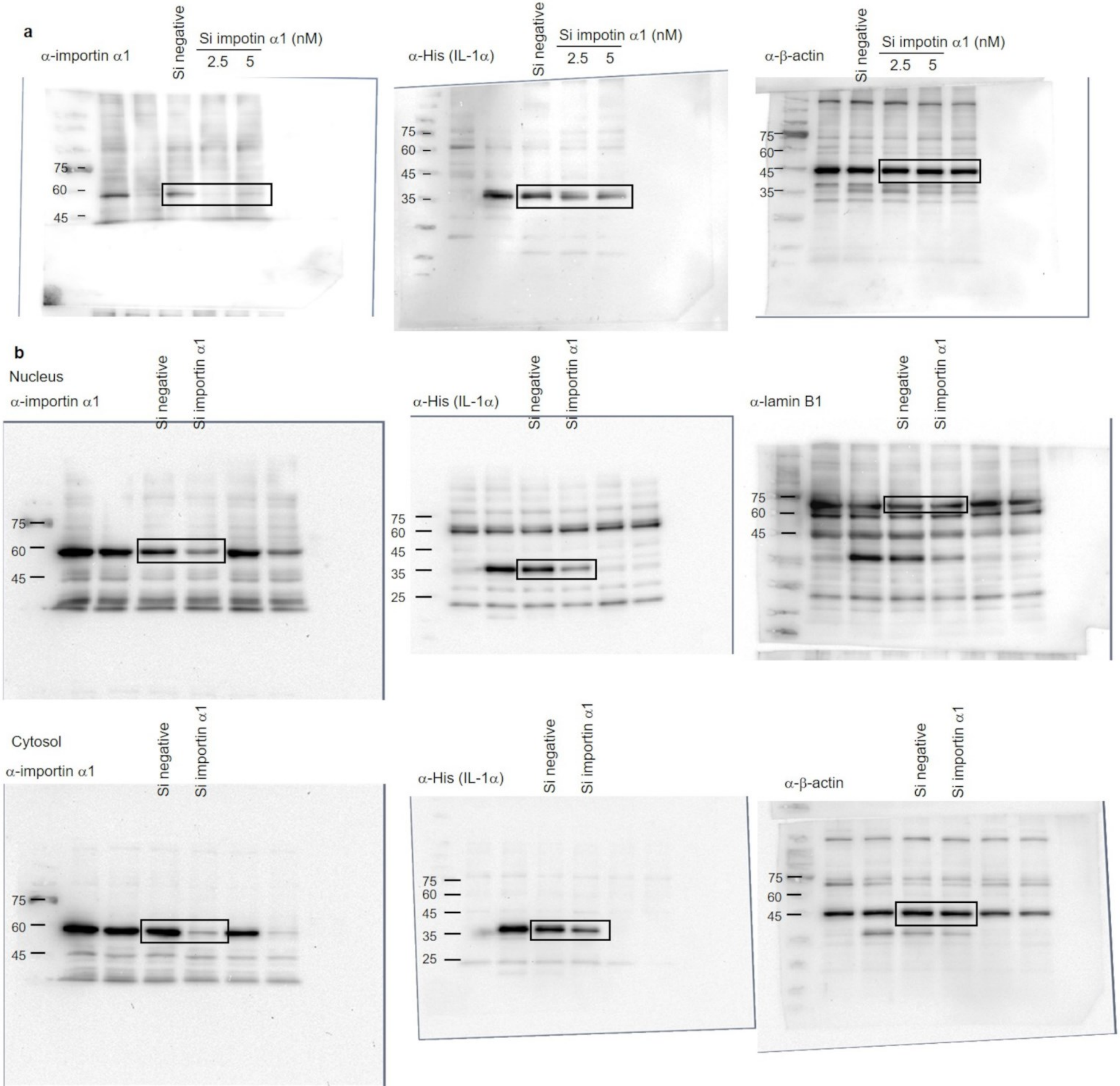
a
α-importin α1
Si negative
Si impotin α1 (nM)
2.5
5
75
60
45
α-His (IL-1α)
Si negative
Si impotin α1 (nM)
2.5
5
75
60
45
35
α-β-actin
Si negative
Si impotin α1 (nM)
2.5
5
75
60
45
35
b
Nucleus
α-importin α1
Si negative
Si importin α1
75
60
45
α-His (IL-1α)
Si negative
Si importin α1
75
60
45
35
25
α-lamin B1
Si negative
Si importin α1
75
60
45
Cytosol
α-importin α1
Si negative
Si importin α1
75
60
45
α-His (IL-1α)
Si negative
Si importin α1
75
60
45
35
25
α-β-actin
Si negative
Si importin α1
75
60
45

Figure 4

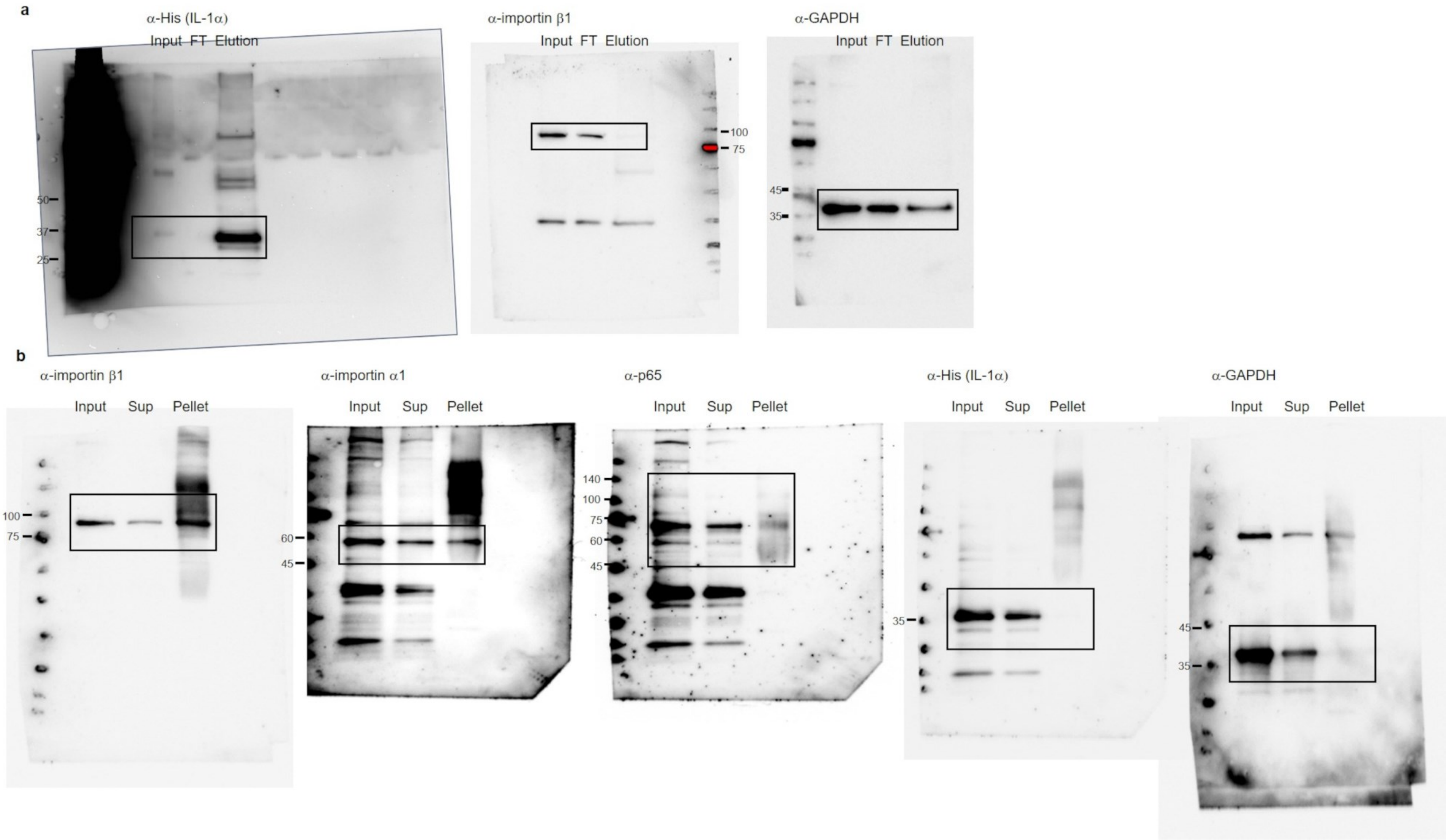
a
α-His (IL-1α)
Input FT Elution
50
37
25
α-importin β1
Input FT Elution
100
75
α-GAPDH
Input FT Elution
45
35
b
α-importin β1
Input Sup Pellet
100
75
α-importin α1
Input Sup Pellet
60
45
α-p65
Input Sup Pellet
140
100
75
60
45
α-His (IL-1α)
Input Sup Pellet
35
α-GAPDH
Input Sup Pellet
45
35

Supplementary figure 1

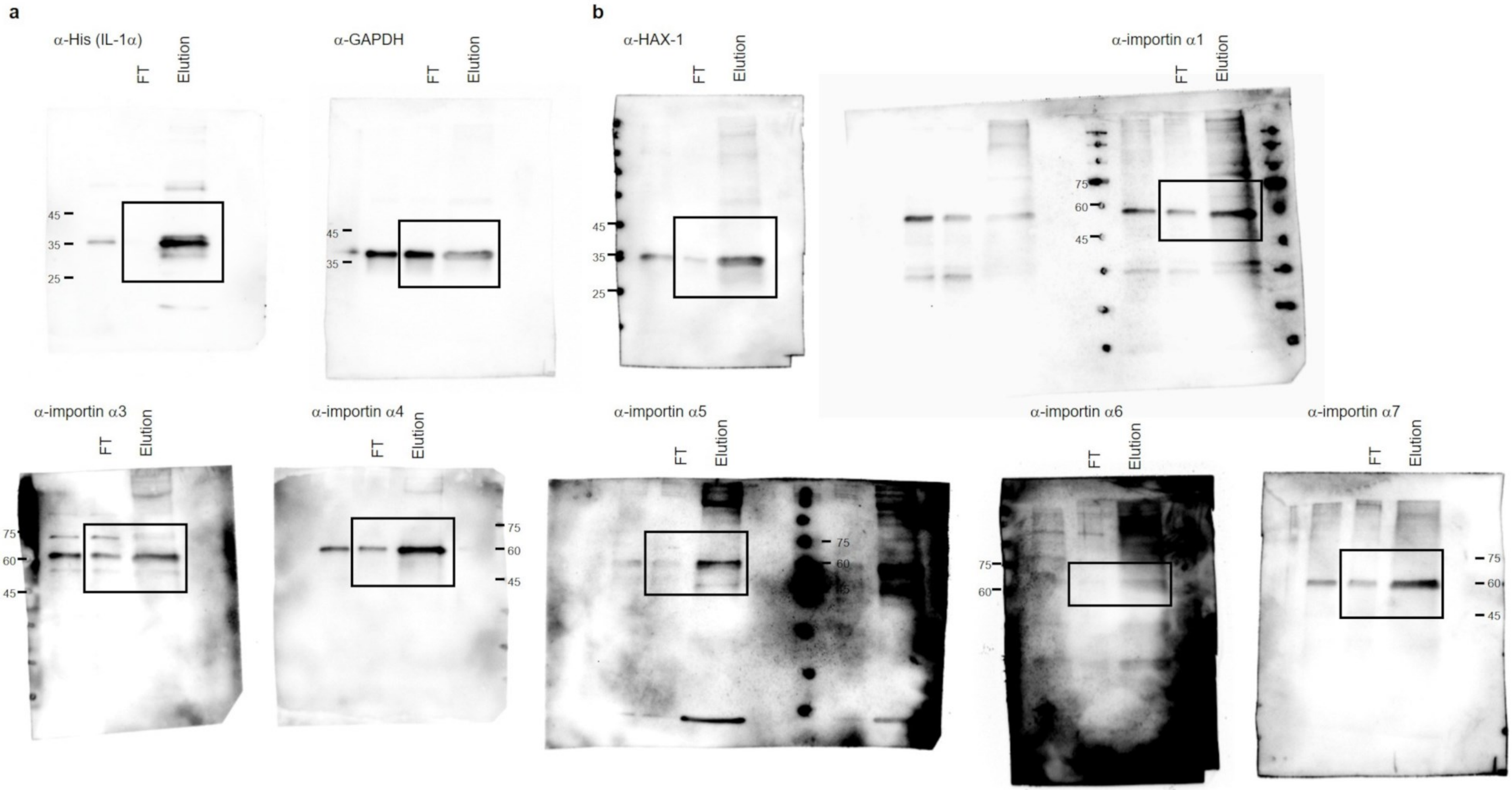

Supplementary figure 2

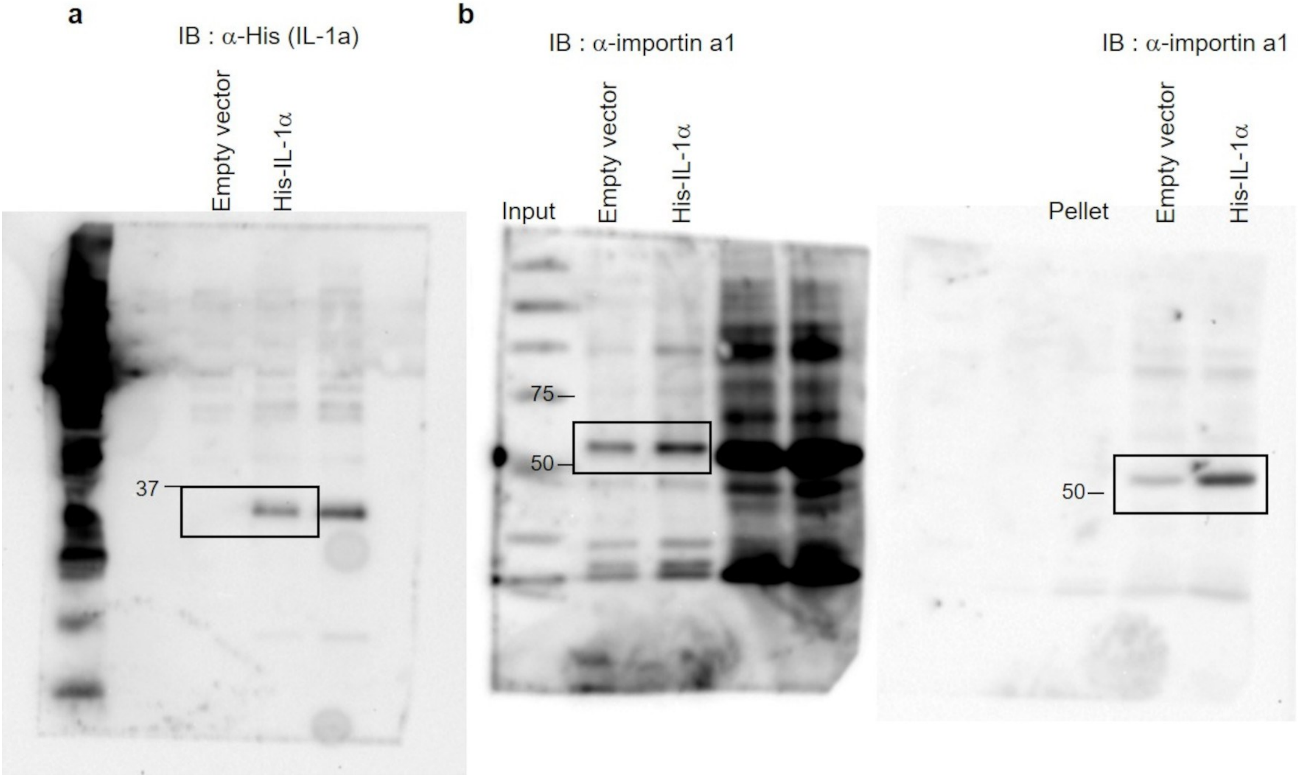

Supplementary figure 3

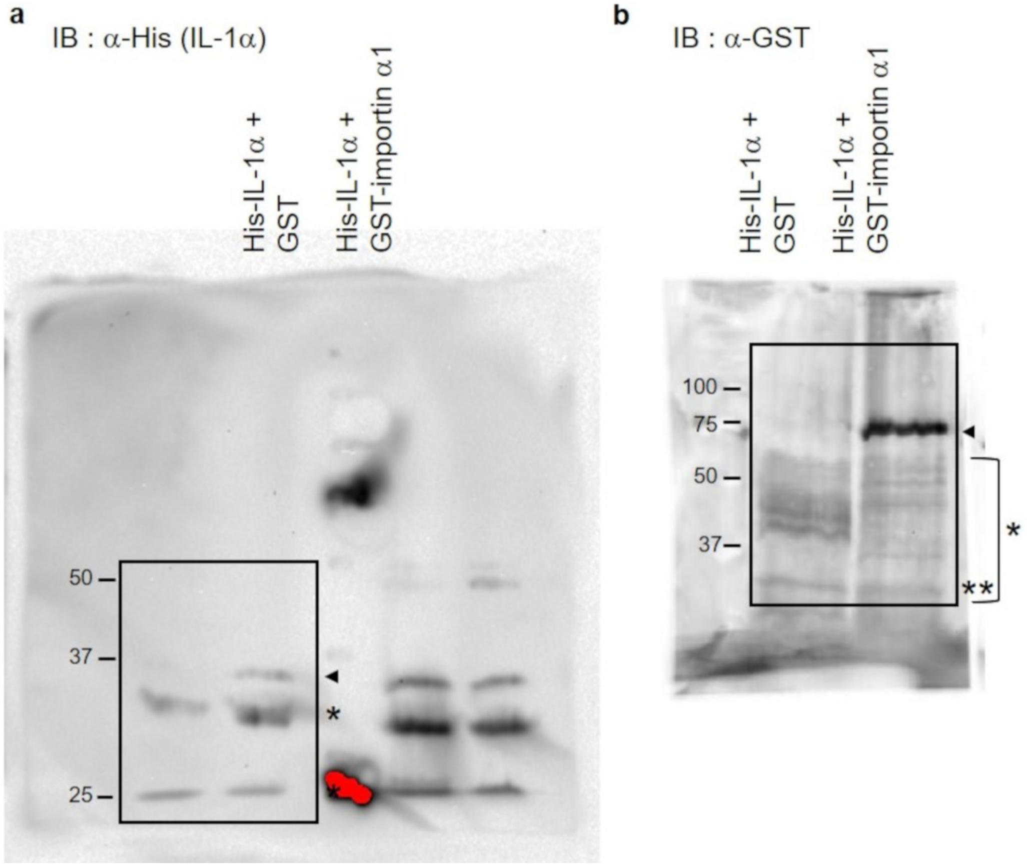

Supplementary figure 4

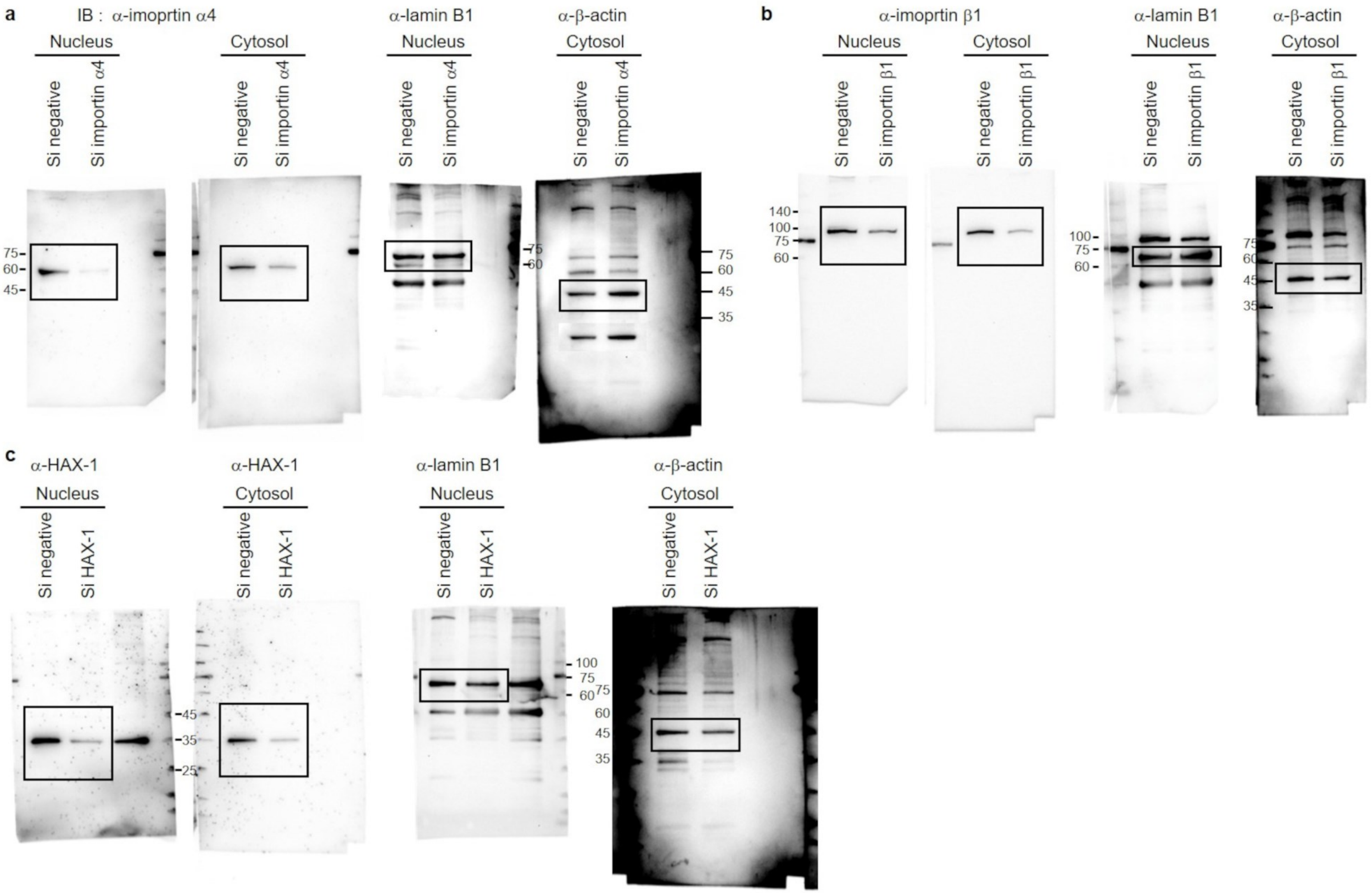

Supplementary figure 4

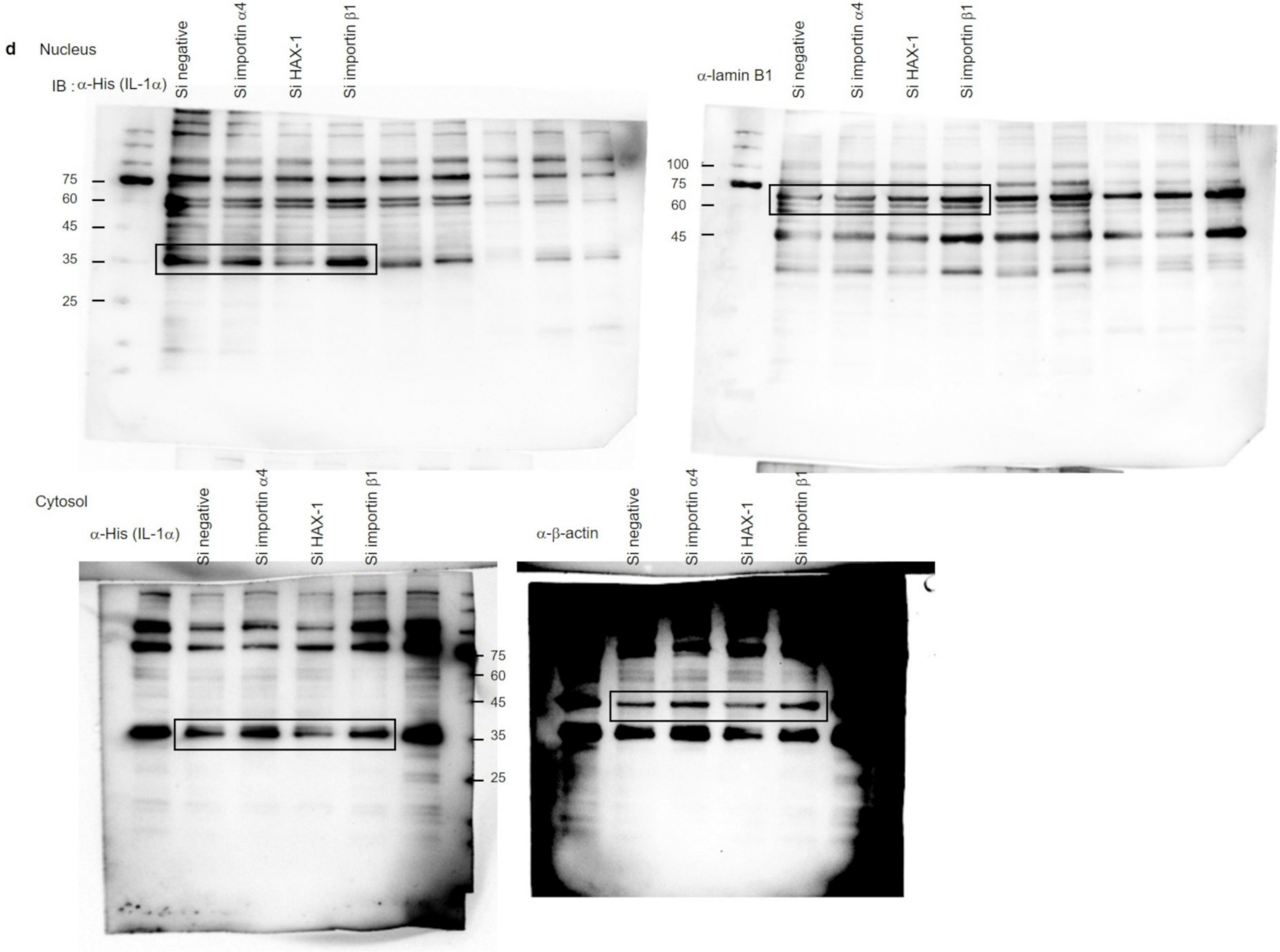

Supplementary figure 4

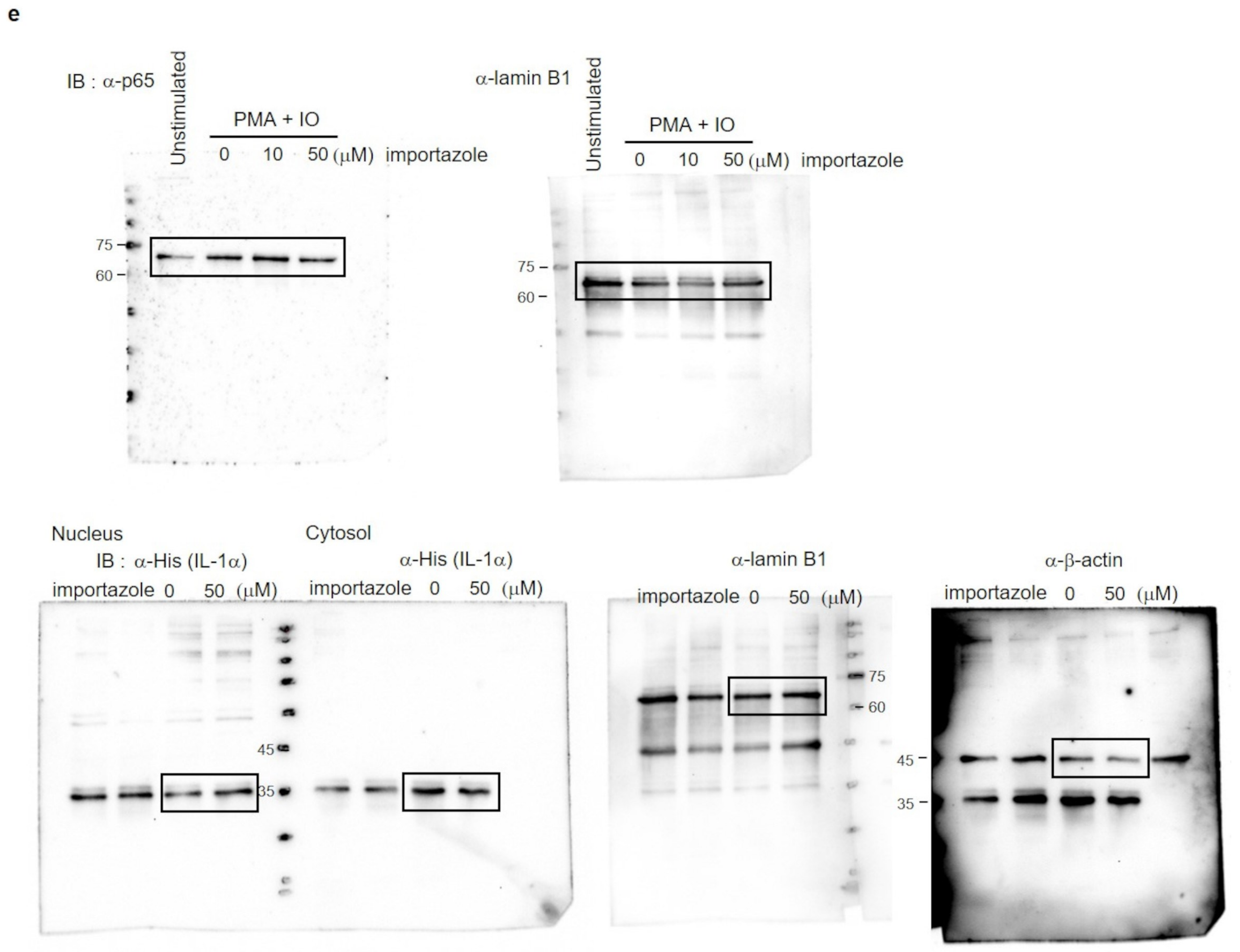

Supplementary figure 5

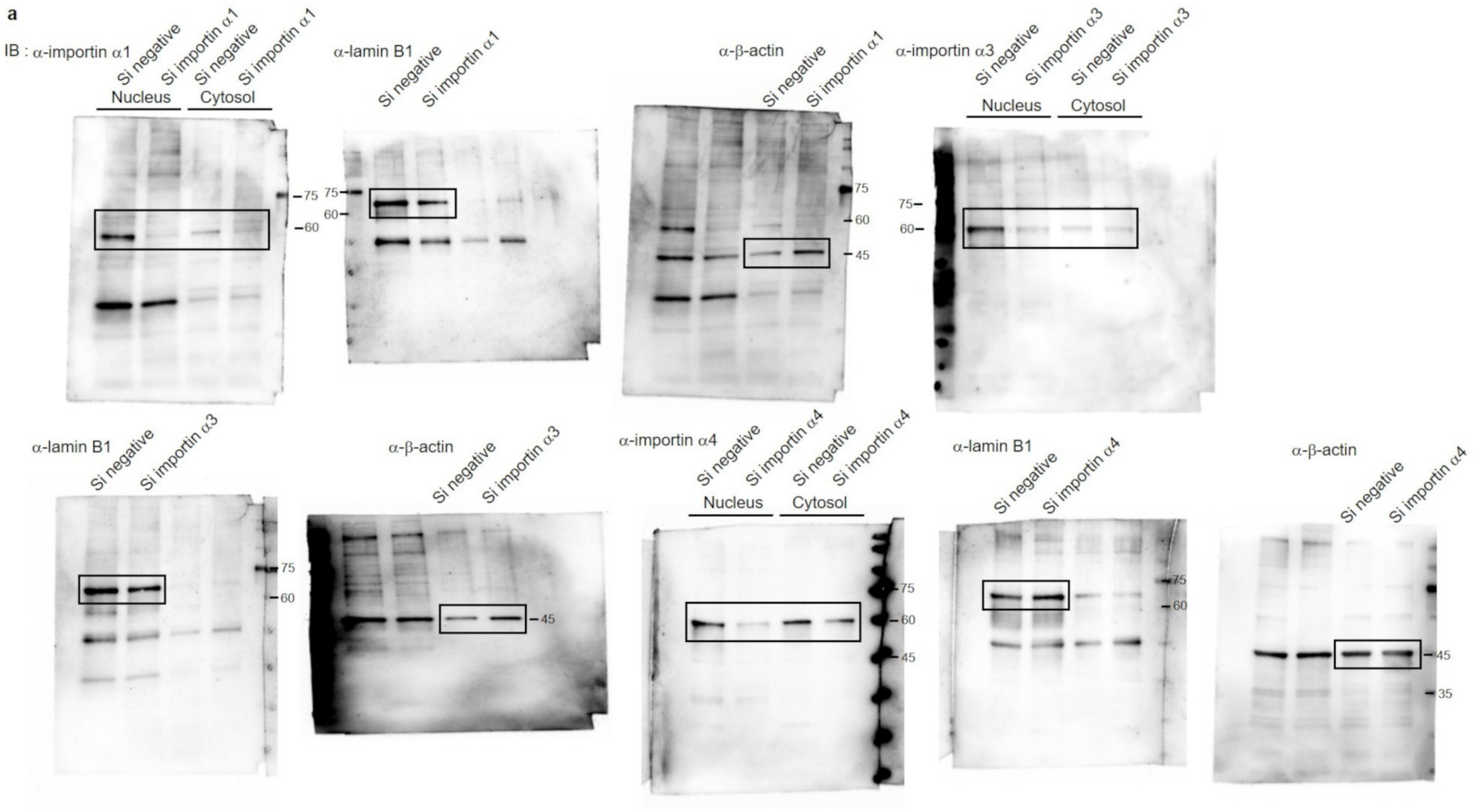

Supplementary figure 5

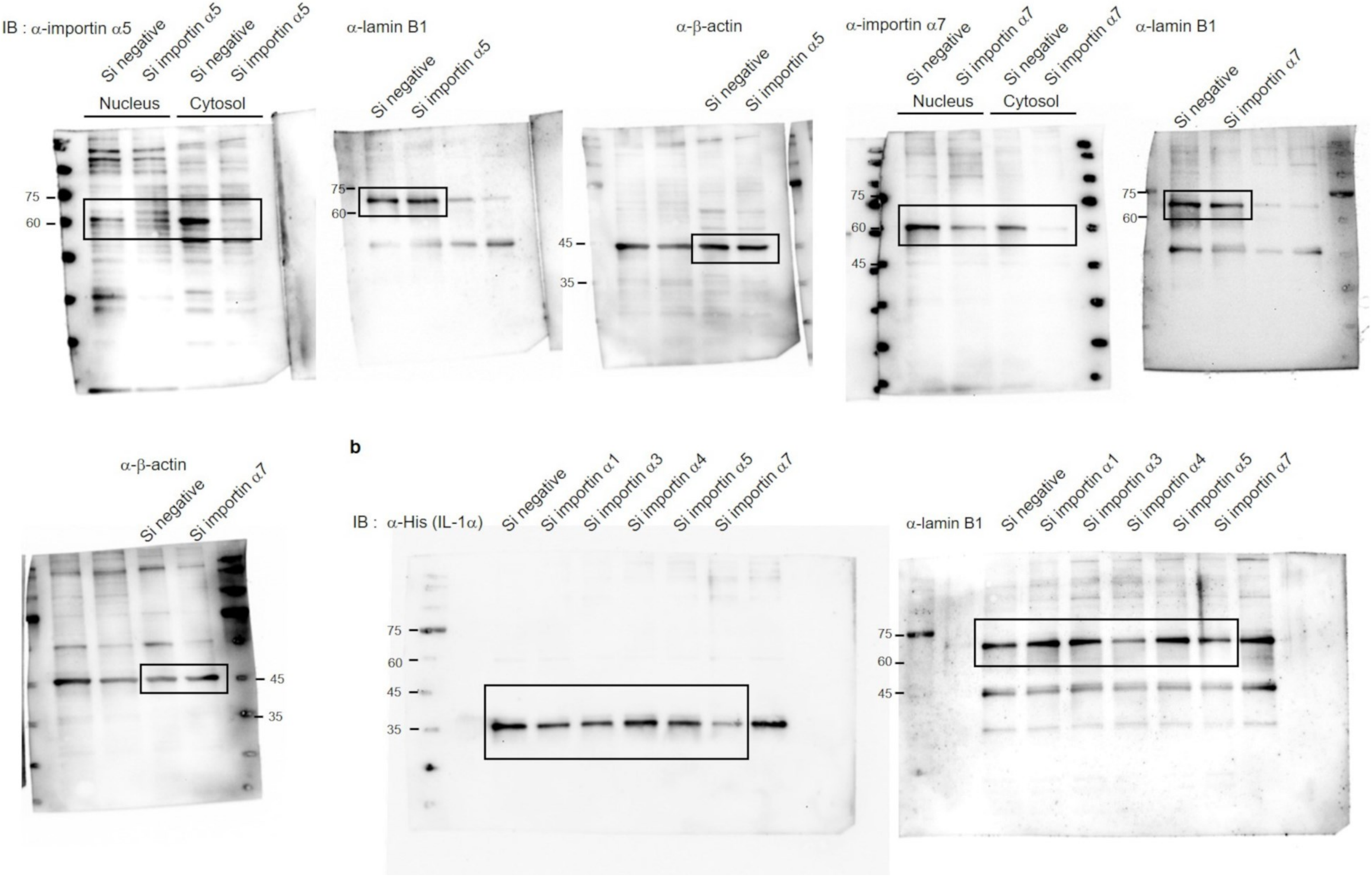

Supplementary figure 6

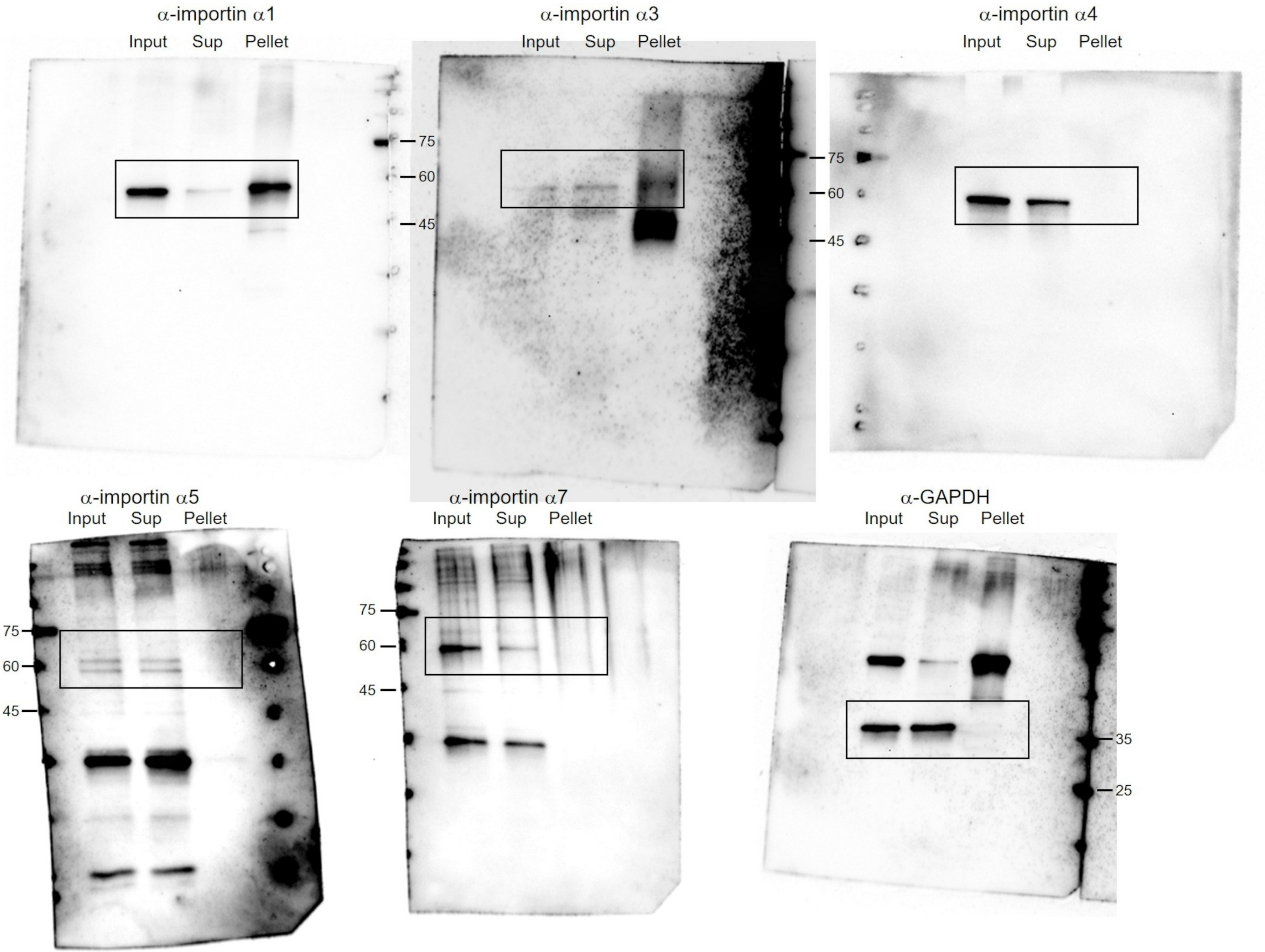

Supplementary figure 7

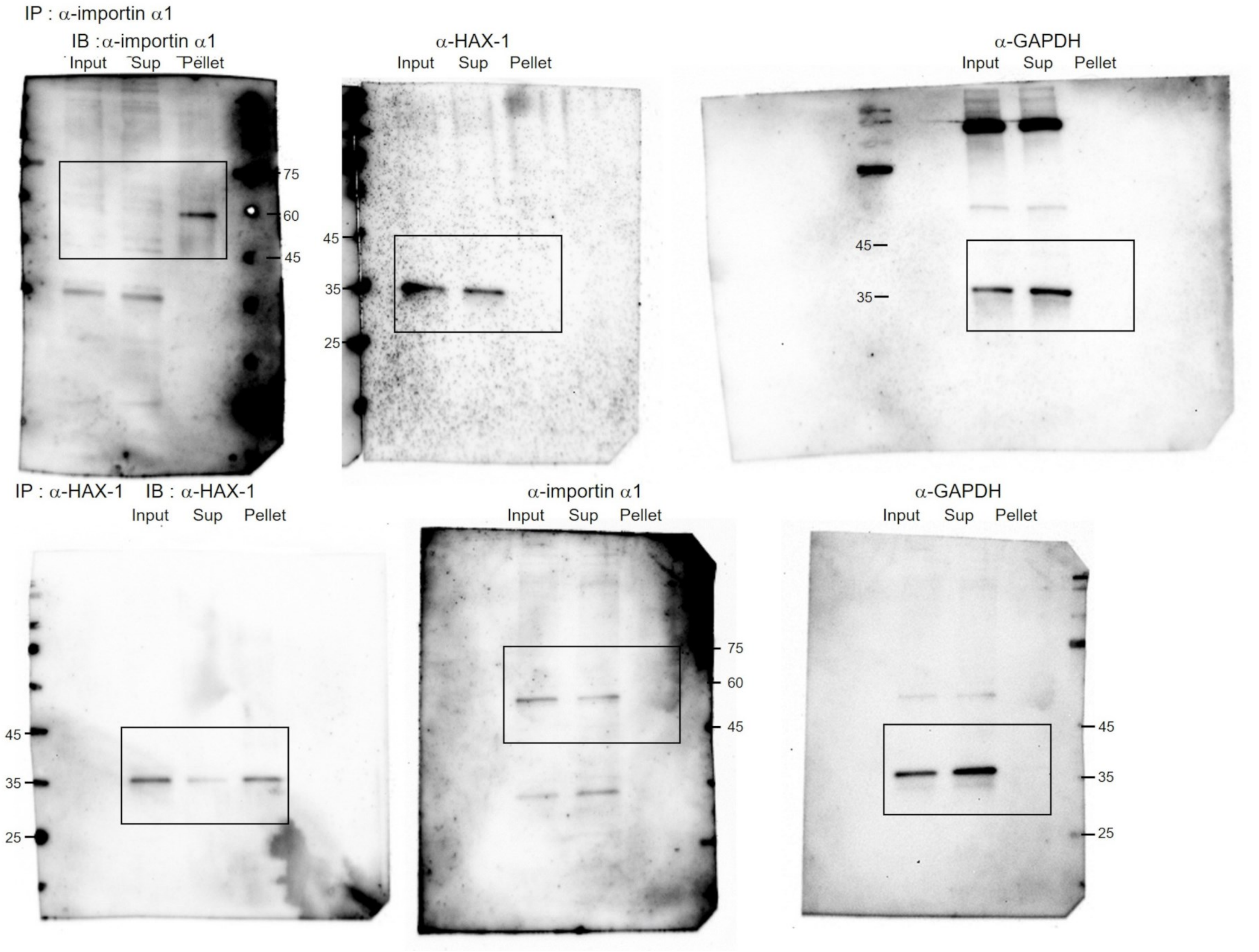

Supplementary figure 8

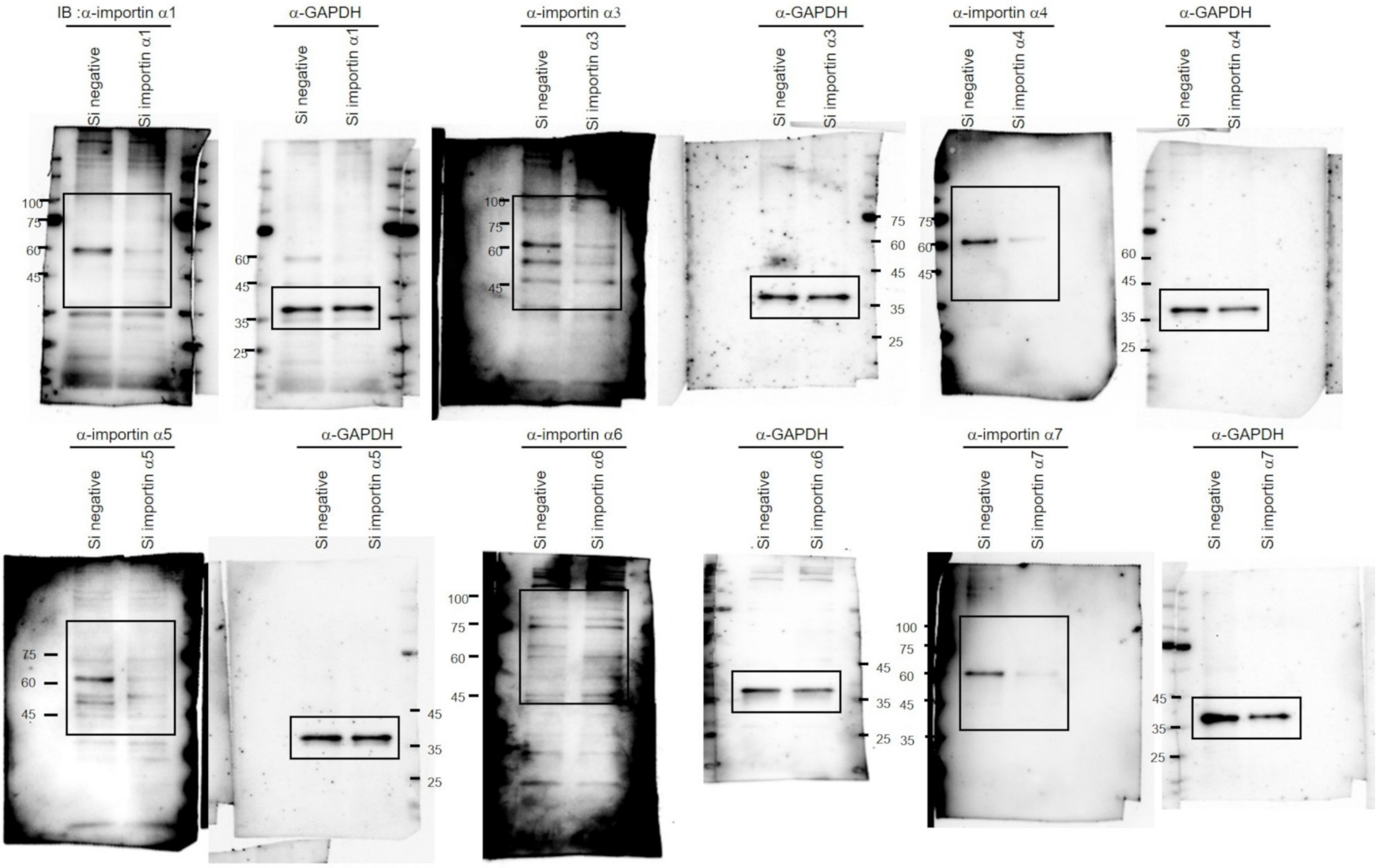

Supplement: Supplementary file 2 — Supplementary Information. [file 41598_2024_51521_MOESM2_ESM.pdf]
