# Supplementary material for: Analysis of the effects of importin α1 on the nuclear translocation of IL-1α in HeLa cells
Source: Sci Rep. 2024 Jan 15;14:1322. doi: 10.1038/s41598-024-51521-w (PMC10789739; doi:10.1038/s41598-024-51521-w)
Supplement: Supplementary file 3 — Supplementary Table 1. [file 41598_2024_51521_MOESM3_ESM.docx]

Supplementary table 1

The primary antibodies against importin α and importin β1
